# Bacterial Genes in the Aphid Genome: Absence of Functional Gene Transfer from *Buchnera* to Its Host

**DOI:** 10.1371/journal.pgen.1000827

**Published:** 2010-02-26

**Authors:** Naruo Nikoh, John P. McCutcheon, Toshiaki Kudo, Shin-ya Miyagishima, Nancy A. Moran, Atsushi Nakabachi

**Affiliations:** 1 Department of Liberal Arts, The Open University of Japan, Chiba, Japan; 2 Center for Insect Science, University of Arizona, Tucson, Arizona, United States of America; 3 Discovery Research Institute, RIKEN, Wako, Saitama, Japan; 4 Advanced Science Institute, RIKEN, Wako, Saitama, Japan; 5 Department of Ecology and Evolutionary Biology, University of Arizona, Tucson, Arizona, United States of America; The University of North Carolina at Chapel Hill, United States of America

## Abstract

Genome reduction is typical of obligate symbionts. In cellular organelles, this reduction partly reflects transfer of ancestral bacterial genes to the host genome, but little is known about gene transfer in other obligate symbioses. Aphids harbor anciently acquired obligate mutualists, *Buchnera aphidicola* (Gammaproteobacteria), which have highly reduced genomes (420–650 kb), raising the possibility of gene transfer from ancestral *Buchnera* to the aphid genome. In addition, aphids often harbor other bacteria that also are potential sources of transferred genes. Previous limited sampling of genes expressed in bacteriocytes, the specialized cells that harbor *Buchnera*, revealed that aphids acquired at least two genes from bacteria. The newly sequenced genome of the pea aphid, *Acyrthosiphon pisum*, presents the first opportunity for a complete inventory of genes transferred from bacteria to the host genome in the context of an ancient obligate symbiosis. Computational screening of the entire *A. pisum* genome, followed by phylogenetic and experimental analyses, provided strong support for the transfer of 12 genes or gene fragments from bacteria to the aphid genome: three LD–carboxypeptidases (*LdcA1, LdcA2*,ψ*LdcA*), five rare lipoprotein As (*RlpA1-5*), *N*-acetylmuramoyl-L-alanine amidase (*AmiD*), 1,4-beta-*N*-acetylmuramidase (*bLys*), DNA polymerase III alpha chain (ψ*DnaE*), and ATP synthase delta chain (ψ*AtpH*). *Buchnera* was the apparent source of two highly truncated pseudogenes (ψ*DnaE* and ψ*AtpH*). Most other transferred genes were closely related to genes from relatives of *Wolbachia* (Alphaproteobacteria). At least eight of the transferred genes (*LdcA1*, *AmiD*, *RlpA1-5*, *bLys*) appear to be functional, and expression of seven (*LdcA1*, *AmiD*, *RlpA1-5*) are highly upregulated in bacteriocytes. The *LdcA*s and *RlpA*s appear to have been duplicated after transfer. Our results excluded the hypothesis that genome reduction in *Buchnera* has been accompanied by gene transfer to the host nuclear genome, but suggest that aphids utilize a set of duplicated genes acquired from other bacteria in the context of the *Buchnera*–aphid mutualism.

## Author Summary

Bacterial lineages have repeatedly evolved intimate symbioses with eukaryotic hosts, the most famous cases being those of the cell organelles, mitochondria, and plastids. Symbiont genomes typically lose many ancestral genes, raising the question of how they function with so few genes. In organelles, part of the answer involves gene transfer to the host genome, allowing maintenance of essential functions. So far, the extent of gene transfer to hosts has not been assessed for other cases of intimate, obligate symbiosis. Aphids harbor an ancient coevolved intracellular symbiont, called *Buchnera*. We used the newly available sequence of the pea aphid genome to conduct an exhaustive computational search for genes of bacterial ancestry. We found that no functional genes have been transferred from *Buchnera*, ruling out such transfer as a driving force in genome reduction in this symbiont. However, the aphid genome does contain eight transcribed genes of apparent bacterial origin, some of which have been duplicated after transfer. Based on their expression patterns, most of these appear to function specifically in the aphid-*Buchnera* symbiosis, presenting the possibility that the maintenance of obligate intracellular symbioses can be affected by the acquisition and duplication of genes transferred from unrelated bacterial lineages.

## Introduction

The smallest known cellular genomes are those of symbiotic bacteria living in insects [1]–[4]. These genomes have lost many genes considered essential in other bacteria, and one proposed explanation is that certain ancestral symbiont genes have been transferred to the host genome, with their products reimported to the symbiont cytosol [1],[5],[6]. This process is known to have occurred in mitochondria and plastids during their evolution as symbiotic associates of eukaryotic cells [7],[8]. Because these associations are mutualistic, selection on host genomes could favor maintenance of genes that benefit the prokaryotic associate. To date, strong evidence for gene transfer from mutualistic symbionts to insect hosts has not been found.

Among the best-known (though not the most extreme) small symbiont genomes are those of *Buchnera aphidicola* (Gammaproteobacteria) (genome size: 420–650 kb), the obligate mutualistic symbiont of aphids [9]–[12]. Aphids are plant-sap sucking insects that have close associations with various microorganisms. Most aphid species, including the pea aphid *Acyrthosiphon pisum*, harbor *Buchnera* within the cytoplasm of specialized cells called bacteriocytes [13]–[13]. Since the initial infection in a common ancestor of aphids more than 100 million years ago [17], *Buchnera* have been subjected to strict vertical transmission through host generations, and the mutualism between *Buchnera* and their host has evolved to the point that neither can reproduce in the absence of the other. *Buchnera* cannot proliferate outside bacteriocytes, and when deprived of *Buchnera*, the host insects suffer retarded growth and sterility, as they are dependent on *Buchnera* for the supply of essential nutrients [15], [18]–[21]. During the course of coevolution with the host, *Buchnera* has lost a number of genes that are considered essential for bacterial existence [9]–[12]. The genome of *Buchnera* from *A*. *pisum* encodes about 620 genes (genome size: 650 kb), which is only one seventh of that of most related bacteria, such as *Escherichia coli* [9]. This raises the question of whether certain genes have been transferred from the genome of ancestral *Buchnera* to the genome of aphids. In addition, aphids often contain other bacterial symbionts and pathogens [16], raising the possibility of LGT from a variety of bacterial lineages. Indeed, evidence is accumulating for extensive transfer of DNA (mostly pseudogenes) from the intracellular bacterium *Wolbachia* (Alphaproteobacteria, Rickettsiales) to its arthropod and nematode hosts [22]–[28]. Moreover, previous studies revealed that *A. pisum* acquired at least two highly transcribed genes from bacteria [29],[30], providing strong evidence that laterally transferred bacterial genes can be of functional importance in metazoan recipients.

Recently, the full genome assembly of *A. pisum* was obtained by the International Aphid Genomics Consortium (IAGC) (IAGC, paper under review). These data provide the first opportunity for an exhaustive search of a genome of an animal that has coevolved with mutualistic intracellular bacteria, including an obligate mutualist with a highly reduced genome. We screened the *A. pisum* genome for bacterial sequences using several computational search strategies, and performed phylogenetic and experimental studies on LGT candidates. We identified a total of 12 genes or gene fragments that seem to have been transferred from bacterial genomes to the genome of an *A. pisum* ancestor. Their structures, phylogenetic positions, evolutionary histories, and expression profiles are further discussed in this paper.

## Results

Our goal was an exhaustive inventory of genes of bacterial origin in the *A. pisum* genome. As is routine for Sanger shotgun sequencing projects, sequences with high identity to the cloning host and vectors were removed as suspected contaminants prior to assembly in the *A. pisum* project. Additionally, sequence reads with high identity to the previously sequenced genome of *Buchnera* str. APS (the *Buchnera* strain derived from *A. pisum*) [9] were removed, since *Buchnera* cells were mixed with host cells in the DNA sample used in the project. For our purpose, these filtered sequences were potential sources of information on LGT. So, as the first step, we retrieved and analyzed them.

### Lack of evidence for LGT from the reads eliminated prior to assembly

Among approximately 4 million sequence reads that were generated for the *A. pisum* genome project, 90,678 reads were removed prior to the assembly of the genome (Acyr_1.0) due to low sequence quality or strong similarities to sequences of *Buchnera*, *E. coli* (cloning host), or the pUC 18 (cloning vector) sequences (IAGC, paper under review). However, if the *A. pisum* genome recently acquired DNA fragments from *Buchnera*, such sequences would show strong similarity to the genomic sequences of *Buchnera*, and may be inappropriately removed at this stage. To assess this possibility, we screened the discarded sequences for LGT candidates using three independent methods.

A single sequence read with regions of similarity to bacterial sequences and invertebrate sequences represents a potential candidate for an *A. pisum* genomic fragment containing laterally transferred bacterial DNAs. To search for such candidates, we first used all of the 90,678 reads as queries, in BLASTX and BLASTN searches conducted against bacterial databases (see Materials and Methods, Table S1). This revealed 33,686 reads with region(s) significantly similar (BLASTX bit score ≥40, BLASTN bit score ≥55) to bacterial sequences (Figure S1, box 1). Subsequently, these 33,686 reads were subjected to BLASTX and BLASTN searches against the RefSeq invertebrate databases (Figure S1, box 2), demonstrating that 19,624 out of 33,686 reads also have region(s) significantly similar (BLASTX bit score ≥40, BLASTN bit score ≥55) to invertebrate sequences. Of these, 19,279 reads contained a single region with similarity to both bacterial and invertebrate sequences; such regions are not related to LGT and instead represent evolutionarily conserved genes, which are widely distributed both in prokaryotes and eukaryotes (Figure S1, box 3). The 345 remaining reads were apparently chimeric and were subjected to BLASTX and BLASTN searches against the National Center for Biotechnology Information (NCBI) non-redundant (nr) database (Figure S1, box 4), and visually inspected one by one. This revealed that 96 reads were parts of pUC 18 or other vectors; these were discarded. An additional 20 reads contained low-complexity sequences (homopolymers or short repeats) and were judged to be insignificant and removed. Because we expect any given genomic region to be covered by at least two high quality reads, we removed 36 singletons showing chimeric bacterial-aphid sequences as potential artifacts introduced by cloning/sequencing errors. The remaining 193 reads showed only weak and unreliable similarity to bacterial or animal sequences, leaving no promising candidates for LGT from this collection of reads.

To further assess this population of precluded reads, we assembled the 90,678 discarded reads using phred/phrap. The assembly produced 5,094 contigs from 38,813 reads, leaving 51,865 reads as singletons. Using these contigs as queries, BLASTX and BLASTN searches were conducted against the bacterial protein database and the *A. pisum* genome assembly (Acyr_1.0), respectively. If a single contig has distinct regions each showing strong similarity to bacterial and *A. pisum* sequences, such a chimeric sequence would be a promising LGT candidate as mentioned above. However, no such contigs were found, again indicating that the precluded 90,678 reads lack promising candidates for LGT.

To further focus on the possibility of recent LGT from *Buchnera*, all 90,678 reads were subjected to BLASTN searches against the *Buchnera* genome from *A. pisum* [*Buchnera* str. APS (NC_002252, NC_002253, NC_002528)] [9]. This revealed 26,529 reads with significant similarity (BLASTN bit score ≥55) to *Buchnera* sequences. After masking the regions similar to *Buchnera*, the sequences were subjected to BLASTN searches against the *A. pisum* genome assembly (Acyr_1.0), revealing 21 reads with regions similar (BLASTN bit score ≥55) to the pea aphid genome. However, none of these reads exhibited features of LGT; that is, they did not exhibit distinct regions with similarity to the *Buchnera* and aphid genomes respectively. Thus, we concluded that the precluded reads contain no evidence for laterally transferred genes.

### Screening of the *A. pisum* genome assembly for LGT candidates

**Figure 1:**
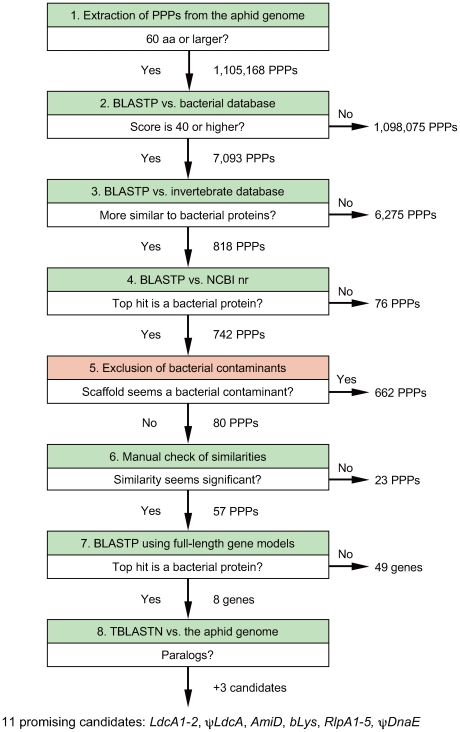
Flow chart of the BLASTP–based screening of the *A. pisum* genome for LGT candidates.

We next focused on screening of the *A. pisum* genome assembly (Acyr_1.0), using three independent strategies designed to detect LGT from any bacterial lineage. Two strategies were based on BLASTP/deduced amino acid sequences (Figure 1) and on BLASTX/six-frame translations (Text S1, Table S2, Figure S2), respectively. These were designed to detect potential transferred genes that might be at different stages of degradation or divergence following transfer to the host genome. We also conducted BLASTN searches designed to detect non-protein-coding sequences transferred from aphid symbionts.

First, all potential polypeptides (PPPs) not less than 60 amino acids were deduced from the genome assembly of *A. pisum* [Acyr_1.0; 22,798 scaffolds (N50 = 86.9 kb; Total size: 464.3 Mb), 6.2× coverage of the 525 Mb *A. pisum* genome] (IAGC, paper under review) as described in the Materials and Methods. A total of 1,105,168 PPPs corresponding to 92,293,525 amino acid residues were obtained (Figure 1, box 1).

Using all 1,105,168 PPPs as queries, BLASTP searches were performed against the bacterial protein database (see Materials and Methods, Table S1). These searches revealed 7,093 PPPs that were significantly similar to bacterial proteins (BLASTP score ≥40) (Figure 1, box 2). Subsequently, these 7,093 PPPs were subjected to BLASTP searches against the RefSeq invertebrate protein database. Comparisons of BLAST hit scores revealed that 818 out of 7,093 PPPs were significantly more similar to bacterial orthologs than to invertebrate orthologs (Figure 1, box 3). To further verify their similarity to bacterial proteins, BLASTP searches were performed against the nr protein database at the NCBI website using the 818 PPPs as queries. For 742 PPPs, top BLAST hits were bacterial proteins (Figure 1, box 4).

### Exclusion of bacterial contaminants

These 742 PPPs were located in 406 scaffolds, most of which were relatively short (<10 kb, whereas N50 of all the *A. pisum* scaffolds is 86.9 kb) and/or contained many unidentified nucleotides (N's). Among them, 331 scaffolds contained only DNA sequences that were nearly identical to bacterial genomic sequences in the non-redundant nucleotide database at NCBI. These 331 scaffolds (Table S3) were assumed to represent bacterial contaminants, and 662 of 742 LGT-candidate PPPs located in these 331 scaffolds were thus eliminated as potential LGT candidates (Figure 1, box 5). Most of the contaminants showed closest matches to related species of Enterobacteriaceae (Gammaproteobacteria) such as members of the genera *Pantoea*, *Serratia*, or *Enterobacter* (Table S3), which are known to infect aphids and other insects as pathogens [31]–[33]. Furthermore, some of these contigs showed near perfect identity to sequences within several BACs sequenced in the *A. pisum* genome project but of clear bacterial origin (AC202220, AC203059, AC203074). As part of the *A. pisum* genome project, 39 of the scaffolds that appeared to derive from bacterial contaminants were screened with PCR in new DNA samples from antibiotic-treated *A. pisum* LSR1 (the sequencing strain), and all were verified to be absent and thus contaminants in the original sample (IAGC, in review).

In addition, two PPPs were located in two distinct scaffolds [SCAFFOLD5147 (EQ115919) and SCAFFOLD7004 (EQ117776)] that appeared to be artifactual chimeric fusions of DNA derived from the genomes of *A. pisum* and bacterial contaminants. In these cases, regions similar to bacterial genes were short (367 nt in the 12,278-nt SCAFFOLD5147 and 373 nt in the 229,440-nt SCAFFOLD7004), almost identical to known bacterial genes (the region in the SCAFFOLD5147 was 87% and 93% identical at the nucleotide and amino acid levels, respectively, to the *fadE* gene (YP_001269130) of *Pseudomonas putida* F1 (Gammaproteobacteria) (CP000712); the region in the SCAFFOLD7004 was 91% and 97% identical at the nucleotide and amino acid levels, respectively, to the *glnD* gene (YP_046710) of *Acinetobacter baumannii* str. SDF (Gammaproteobacteria) (CU468230), and covered only by a single sequence read each (based on visual inspections of the NCBI trace archive). As these scaffolds seemed highly likely to be artifacts due to cloning and/or assembly errors, we also discarded these two PPPs (Figure 1, box 5).

### Exclusion of PPPs weakly similar to bacterial proteins

Twenty of the 80 remaining LGT-candidate PPPs showed only weak similarity to bacterial proteins in the NCBI nr protein database (bit score ≤45 and E-value ≥0.001). Manual inspection of the BLAST hit sequences revealed that each of the aligned regions was short and that hits were derived from various genes that are not related to one another, indicating that the results were not reliable. Thus, these PPPs were also discarded (Figure 1, box 6). In addition, three PPPs showed moderate similarity to bacterial sequences (bit score >50, E-value <0.0001), but the aligned regions of both the queries and hit sequences consisted of tandem repeat sequences. As lengths of the repeat units of the queries and hit sequences were different and the similarity appeared to be detected only by chance, these PPPs were also removed from the LGT candidates (Figure 1, box 6).

### Exclusion of PPPs that were parts of proteins with higher similarity to metazoan proteins

Fifty-four of the 57 remaining LGT-candidate PPPs were parts of the *A. pisum* proteins predicted by the NCBI and IAGC. Using full-length amino acid sequences of 54 corresponding proteins as query sequences, BLASTP searches were performed against nr protein database at NCBI. Forty-nine of the 54 proteins were more similar to animal proteins than to bacterial proteins, and were orthologs of proteins widely distributed both in prokaryotes and eukaryotes (Figure 1, box 7). Only a fraction of each PPP showed slightly higher similarity (BLAST bit score difference <13) to bacterial proteins than to animal proteins. Thus, none of these 49 proteins appeared more similar to bacterial proteins than to animal proteins, and so were removed from the LGT-candidates (Figure 1, box 7).

### Promising candidates of LGT

Finally, eight genes corresponding to the eight remaining PPPs were judged as promising LGT candidates. These eight contained two copies of LD-carboxypeptidase (*LdcA*), three copies of rare lipoprotein A (*RlpA*), and one copy each of *N*-acetylmuramoyl-L-alanine amidase (*AmiD*), 1,4-beta-*N*-acetylmuramidase (*bLys*), and DNA polymerase III alpha chain (ψ*DnaE*). To check the presence/absence of more paralogs for these genes, TBLASTN searches were performed against the *A. pisum* genome assembly using deduced amino acid sequences of the eight candidates as queries (Figure 1, box 8). This detected one more *LdcA* and two more *RlpA*s.

We also performed a screen based on six-frame translations of the *A. pisum* genome (BLASTX), which is potentially more sensitive in detecting shorter and degenerate sequences, as the method is not limited by the threshold of the PPP length (≥60 aa) and will produce protein alignments across stop codons (Text S1, Table S2, Figure S2). This method identified 10 of the 11 LTG candidates found in the search based on PPPs, verifying the effectiveness of the two strategies. The BLASTX-based approach identified a single additional candidate, ATP synthase delta chain (ψ*AtpH*).

We also performed BLASTN searches using the genomes of *Buchnera* str. APS (NC_002252, NC_002253, NC_002528) [9] and *Hamiltonella defensa* (NC_012751, NC_012752) (Gammaproteobacteria; a facultative symbiont of aphids) [34] as queries, as such searches could reveal transfers of non-protein-coding fragments that would not have been evident in the PPP-based or the BLASTX-based searches described above. However, no additional LGT candidates were obtained in these searches.

Thus, in total, computational screens identified 12 promising LGT candidates (*LdcA1, LdcA2*,ψ *LdcA*, *AmiD*, *bLys*, *RlpA1*, *RlpA2*, *RlpA3*, *RlpA4*, *RlpA 5*, ψ*DnaE*, and ψ*AtpH*) in the *A. pisum* genome (Table 1). One each of *LdcA*s (now renamed *LdcA1*, ACYPI009109) and *RlpA*s (renamed *RlpA4*, ACYPI004737) were originally detected in our previous transcriptome analysis of the *A. pisum* bacteriocyte [29], and were further verified to have been transferred from bacteria to the aphid genome via LGT [30]. Extant *Buchnera* lacks these genes other than *dnaE* and *atpH* [9], whereas many other bacteria, including *E. coli*, a close relative of *Buchnera*, possess all of them [35]. To further verify the presence of these genes in the *A. pisum* genome, we conducted experimental analyses using quantitative PCR.

**Table 1:** LGT candidates in the *A. pisum* genome.

Gene	Gene name	Gene ID	Scaffold	Position		Top BLAST hit*		
symbol			ID	Start	End	Species	Score	E-value
*LdcA1*	LD-carboxypeptidase_1	ACYPI009109	EQ117656 (SCAFFOLD6884)	3658	2295	*Wolbachia* endosymbiont of *Culex quinquefasciatus* Pel	310	2E-82
ψ*LdcA*	LD-carboxypeptidase (pseudo)	N/A	EQ122282 (SCAFFOLD11510)	81202	80565	*Wolbachia* endosymbiont of *Drosophila melanogaster*	99	2E-19
*LdcA2*	LD-carboxypeptidase_2	N/A	EQ111801 (SCAFFOLD1029)	7224	10729	N/A	N/A	N/A
*AmiD*	*N*-acetylmuramoyl-L-alanine amidase	ACYPI006531	EQ126042 (SCAFFOLD15270)	18473	11268	*Orientia tsutsugamushi*	220	1E-55
*bLys*	1,4-beta-*N*-acetylmuramidase	ACYPI004424	EQ113280 (SCAFFOLD2508)	40946	58233	*Wolbachia* sp. *w*Ri	214	3E-54
*RlpA1*	Rare lipoprotein A_1	AUG4_SCAFFOLD5510.g2.t1**	EQ116281 (SCAFFOLD5509)	9126	11255	*Leptospirillum* sp. Group II UBA	54	3E-06
*RlpA2*	Rare lipoprotein A_2	ACYPI008496	EQ116281 (SCAFFOLD5509)	22801	17534	*Pelobacter carbinolicus*	47	7E-04
*RlpA3*	Rare lipoprotein A_3	ACYPI38879	EQ116281 (SCAFFOLD5509)	33213	30611	*Rhodopseudomonas palustris* TIE-1	55	3E-06
*RlpA4*	Rare lipoprotein A_4	ACYPI004737	EQ116281 (SCAFFOLD5509)	34287	37803	*Bradyrhizobium* sp. BTAi1	84	1E-14
*RlpA5*	Rare lipoprotein A_5	ACYPI005979	EQ116281 (SCAFFOLD5509)	42895	40402	*Desulfonatronospira thiodismutans* ASO3-1	49	3E-04
ψ*DnaE*	DNA polymerase III alpha chain (pseudo)	N/A	EQ126219 (SCAFFOLD15447)	10176	10500	*Buchnera aphidicola* str. APS	82	4E-19
ψ*AtpH*	ATP synthase delta chain (pseudo)	N/A	EQ115356 (SCAFFOLD4584)	9331	9630	*Buchnera aphidicola* str. APS	79	1E-13

*: BLASTX for ψDnaE and ψAtpH. BLASTP for the rest.

**: The name of this gene model is based on the older version of the scaffold ID.

### Quantitative PCR verified the presence of LGT candidates in the *A. pisum* genome

Bacterial symbionts, contaminants and pathogens present within the host are not expected to be at constant copy number relative to host genome copies, when multiple tissues or hosts are examined. For example, *Buchnera* and facultative symbionts show large fluctuations in genome and cell copy number relative to single copy *A. pisum* genes, depending on the tissue sampled and on the age and condition of the individual aphid (e.g., [36]–[38]). Pathogens are expected to vary even more in abundance, and typically are entirely absent from aphids, based on PCR assays [31]. In contrast, sequences that are part of the host genome will display nearly the same copy number as single copy genes from the genome, both reflecting the number of host genomic copies within a sample.

**Figure 2:**
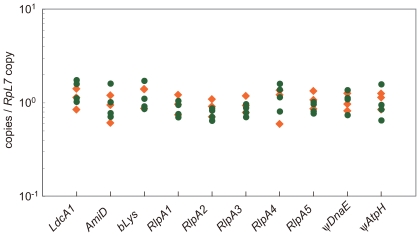
Copy numbers of LGT candidates in the *A. pisum* genome. Orange rhombi, copy numbers in the strain LSR1 (n = 3); Green circles, copy numbers in the strain ISO (n = 4). The copy numbers are shown in terms of copies of target genes per copy of the standard gene, *RpL7*. All quantitative PCRs were performed in triplicate. Each data point thus shows the mean of three separate quantitative PCRs.

To distinguish between the hypotheses that LGT candidates derive from the aphid genome rather than from contaminants, we examined copy number of these genes relative to a known single copy gene in the aphid genome, using real time quantitative PCR (Figure 2). Two *A. pisum* strains were used for the analysis; one was the strain LSR1 (n = 3), the North American strain that was used for the genome sequencing, and the other was the strain ISO (n = 4), the Japanese strain that was used for our previous transcriptome analysis of the bacteriocyte [29],[30]. A ribosomal protein gene, *RpL7*, which is believed to be present as a single copy per haploid *A. pisum* genome, was used as a standard. (This gene is present in only one copy in the *A. pisum* genome project and is only known as a single copy gene in other genomes.) Of the three *LdcA*s, only *LdcA1* was analyzed. The target/standard ratios (mean ± SE) for *LdcA1*, *AmiD*, *bLys*, *RlpA1*, *RlpA2*, *RlpA3*, *RlpA4*, *RlpA5*, ψ*DnaE*, and ψ*AtpH* were 1.27±0.12, 0.98±0.13, 1.18±0.13, 0.91±0.07, 0.82±0.06, 0.89±0.06, 1.16±0.13, 0.98±0.07, 1.05±0.09, and 1.04±0.12, respectively (Figure 2). That these ratios were nearly constant across samples and centered around 1 (p>0.05, one-way ANOVA followed by Tukey-Kramer test) strongly suggests that they are encoded in the *A. pisum* genome as single-copy genes. Moreover, the ratios for the nine genes showed no significant difference between the two *A. pisum* strains (p>0.05, Student's *t*-test), indicating that both strains encode these genes in their genomes. These results are a strong indicator that the candidate genes do not derive from contaminant bacteria, as the titer of such contaminants would dramatically differ among aphid individuals, which should result in ratio variation among samples.

### Aphids appear to have acquired only ψ*DnaE* and ψ*AtpH* from *Buchnera*

To further characterize these genes, we performed detailed structural and molecular phylogenetic analyses. The candidate in SCAFFOLD15447 (EQ126219) was similar to bacterial genes encoding DNA polymerase III alpha subunit (DnaE) (Table 1). The top BLASTX hit was DNA polymerase III alpha subunit [*Buchnera aphidicola* str. APS] (NP_240067.1) (E = 4×10^−19^), and essentially all the subordinate hits were DNA polymerase III alpha subunit proteins of various lineages of bacteria. The amino acid sequence of the aphid DnaE was 66% and 38% identical to DnaE orthologs of *Buchnera* str. APS and *E. coli* K12, respectively (Figure S3).

**Figure 3:**
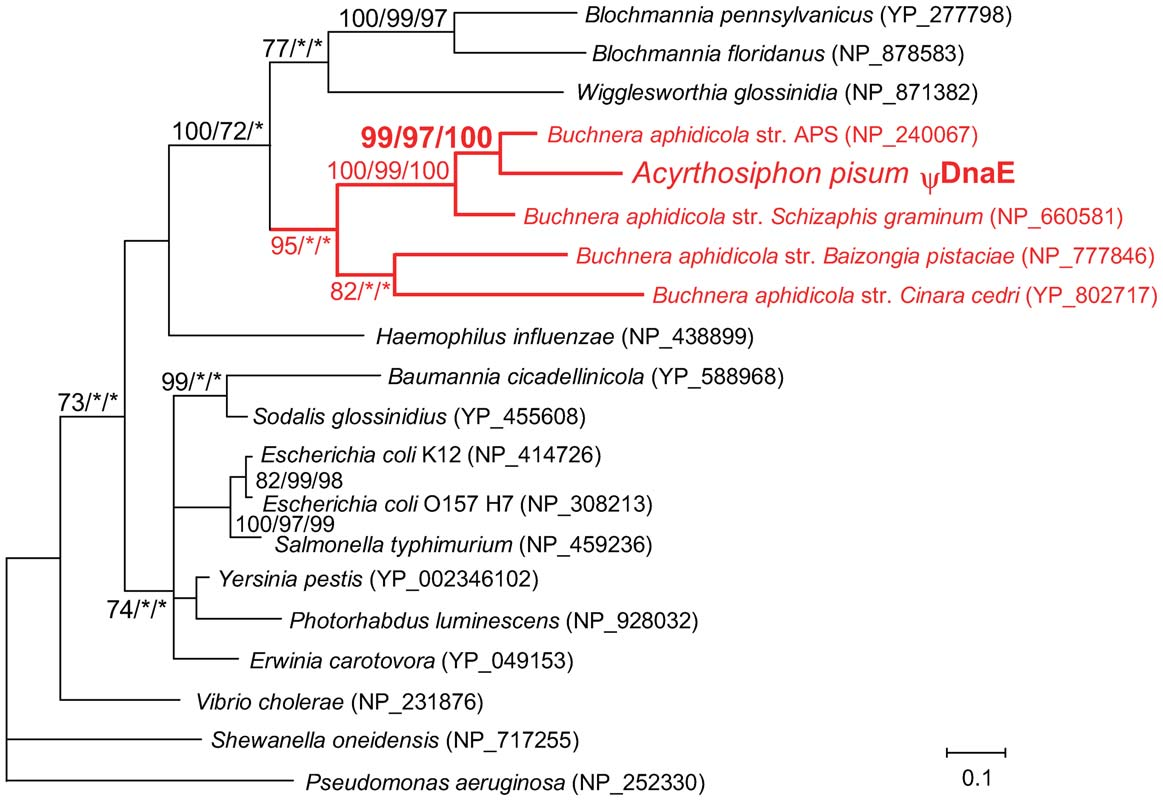
Phylogenetic position of the aphid ψ*DnaE*. A total of 90 aligned amino acid sites were subjected to the analysis. Orthologs from Gammaproteobacteria were used, as BLAST searches indicated that all top hits were in this group. A Bayesian tree is shown; the ML tree and NJ tree exhibited substantially the same topologies. On each node, support values over 50 are shown (BI/ML/NJ). Asterisks (*) indicate support values lower than 50. The *A. pisum*-*Buchnera* cluster is shown in red. Accessions of the sequences are shown in parentheses. Scale bar indicates substitutions per site.

Phylogenetic analyses clearly showed that the *A. pisum* ψDnaE forms a monophyletic clade with DnaE of *Buchnera* str. APS (99% in Bayesian inference (BI), 97% in maximum likelihood (ML), 100% in neighbor-joining (NJ)), which is sister to that of *Buchnera* str. *Schizaphis graminum* (the strain derived from another aphid species, *S. graminum*) (100/99/100) (Figure 3). This indicates that *A. pisum* relatively recently acquired ψ*DnaE* from *Buchnera*, after its divergence from the lineage leading to *S. graminum* (50–70 million years ago) [10],[17]. However, the predicted aphid DnaE was 120 aa in length, whereas the DnaE of *Buchnera* str. APS is 1,161 aa, the approximate length of this gene in bacteria generally. No other DNA sequence corresponding to the missing part of DnaE was found in the *A. pisum* genome assembly. These observations imply that the *A. pisum* ψ*DnaE* is a pseudogene. We further confirmed this possibility using a relative rate test showing that the *A. pisum* copy evolves at an accelerated rate, as expected for a pseudogene (Text S2).

The candidate in the SCAFFOLD4584 (EQ115356) was similar to bacterial genes encoding ATP synthase delta subunit (AtpH) (Table 1). The top BLASTX hit was ATP synthase delta subunit [*Buchnera* str. APS] (NP_239847.1) (E = 1×10^−13^), and essentially all the subordinate hits were ATP synthase delta subunit proteins of various lineages of bacteria. The amino acid sequence of aphid AtpH was 58% and 35% identical to AtpH orthologs of *Buchnera* str. APS and *E. coli* K12, respectively (Figure S4). However, the predicted aphid AtpH was 100 aa in length, and has three intermittent stop codons, whereas the AtpH of *Buchnera* str. APS is 177 aa, the approximate length of this gene in bacteria generally. No other DNA sequence corresponding to the missing part of AtpH was found in the *A. pisum* genome assembly. These observations imply that the *A. pisum* ψ*AtpH* is also a pseudogene.

**Figure 4:**
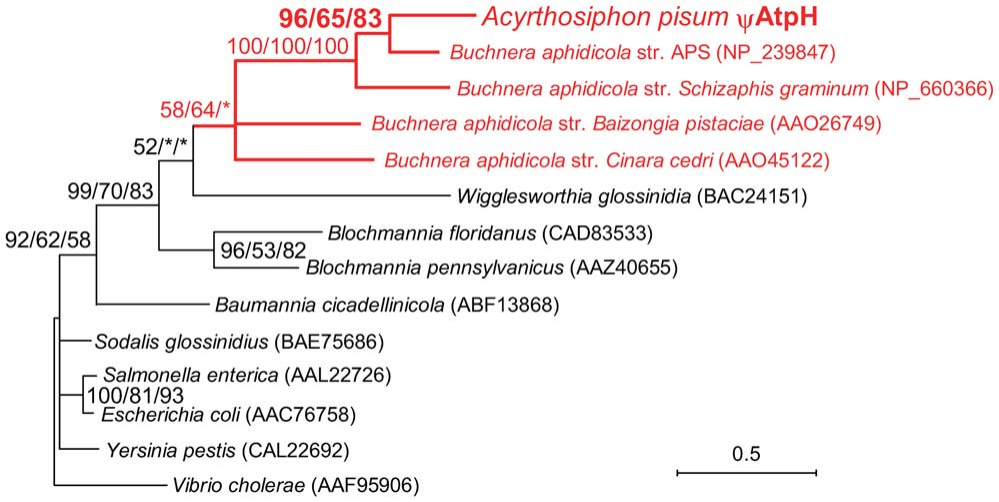
Phylogenetic position of the aphid ψ*AtpH*. A total of 95 aligned amino acid sites were subjected to the analysis. Orthologs from Gammaproteobacteria were used, as BLAST searches indicated that all top hits were in this group. A Bayesian tree is shown; the ML tree and NJ tree exhibited substantially the same topologies. On each node, support values over 50 are shown (BI/ML/NJ). Asterisks (*) indicate support values lower than 50. The *A. pisum*-*Buchnera* cluster is shown in red. Accessions of the sequences are shown in parentheses. Scale bar indicates substitutions per site.

Phylogenetic analyses gave results for the *A. pisum* ψ*AtpH* that were similar to those for ψ*DnaE*. The copy in the *A. pisum* genome forms a clade with AtpH of *Buchnera* str. APS (96% in BI, 65% in ML, 83% in NJ), which is sister to that of *Buchnera* str. *S. graminum* (100% in BI, ML, and NJ) (Figure 4). Thus, *A. pisum* relatively recently acquired both ψ*AtpH* and ψ*DnaE* from *Buchnera*, after divergence from a common ancestor of *A. pisum* and *S. graminum*.

### Aphid *LdcA* was duplicated after LGT from a bacterium

**Figure 5:**
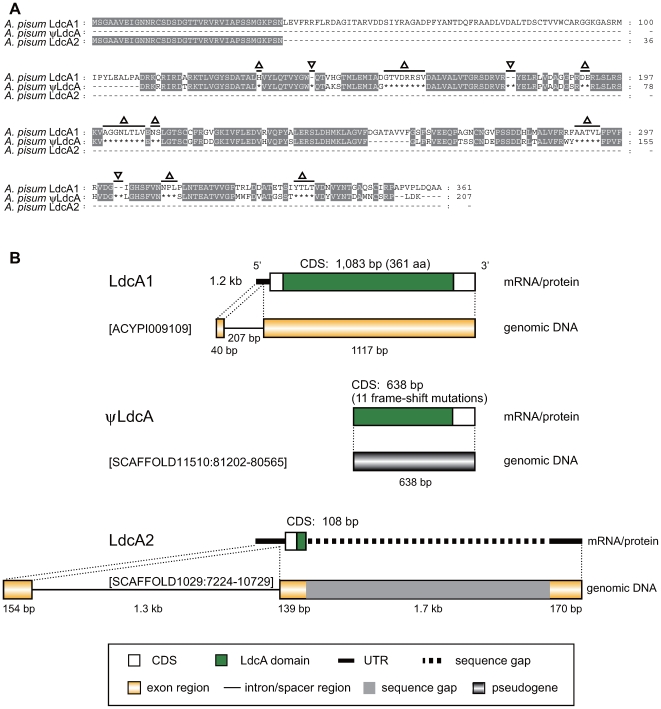
Structure of the aphid LdcAs. (A) Alignment of amino acid sequences of LdcAs. Residues conserved in two lineages are shaded gray. Triangles and reverse-triangles indicate frameshift deletions and insertions, respectively, in ψ*LdcA*. Dashes (-) indicate alignment gaps. Asterisks (*) indicate gaps caused by frameshifts. (B) Domain structures of the aphid LdcA proteins and structures of the corresponding mRNAs and genomic DNAs.

Three [ACYPI009109, SCAFFOLD11510 (EQ122282) nucleotide number: 81202.80565, and SCAFFOLD1029 (EQ111801) nucleotide number: 7224.10729] (Table 1) of the 12 candidates were similar to bacterial *ldcA* genes, which encodes LD-carboxypeptidases that are required for recycling murein (peptidoglycan), a component of the bacterial cell wall [39]. As demonstrated previously [30], one of the *A. pisum LdcA* genes [*LdcA1*; ACYPI009109 in the SCAFFOLD6884 (EQ117656)] has a functional protein-coding sequence. On the other hand, another gene (ψ*LdcA* in the SCAFFOLD11510) newly found in this study (Table 1) had 11 frame-shift mutations in its potential coding sequence (Figure 5), suggesting that this copy of *LdcA* is a pseudogene.

**Figure 6:**
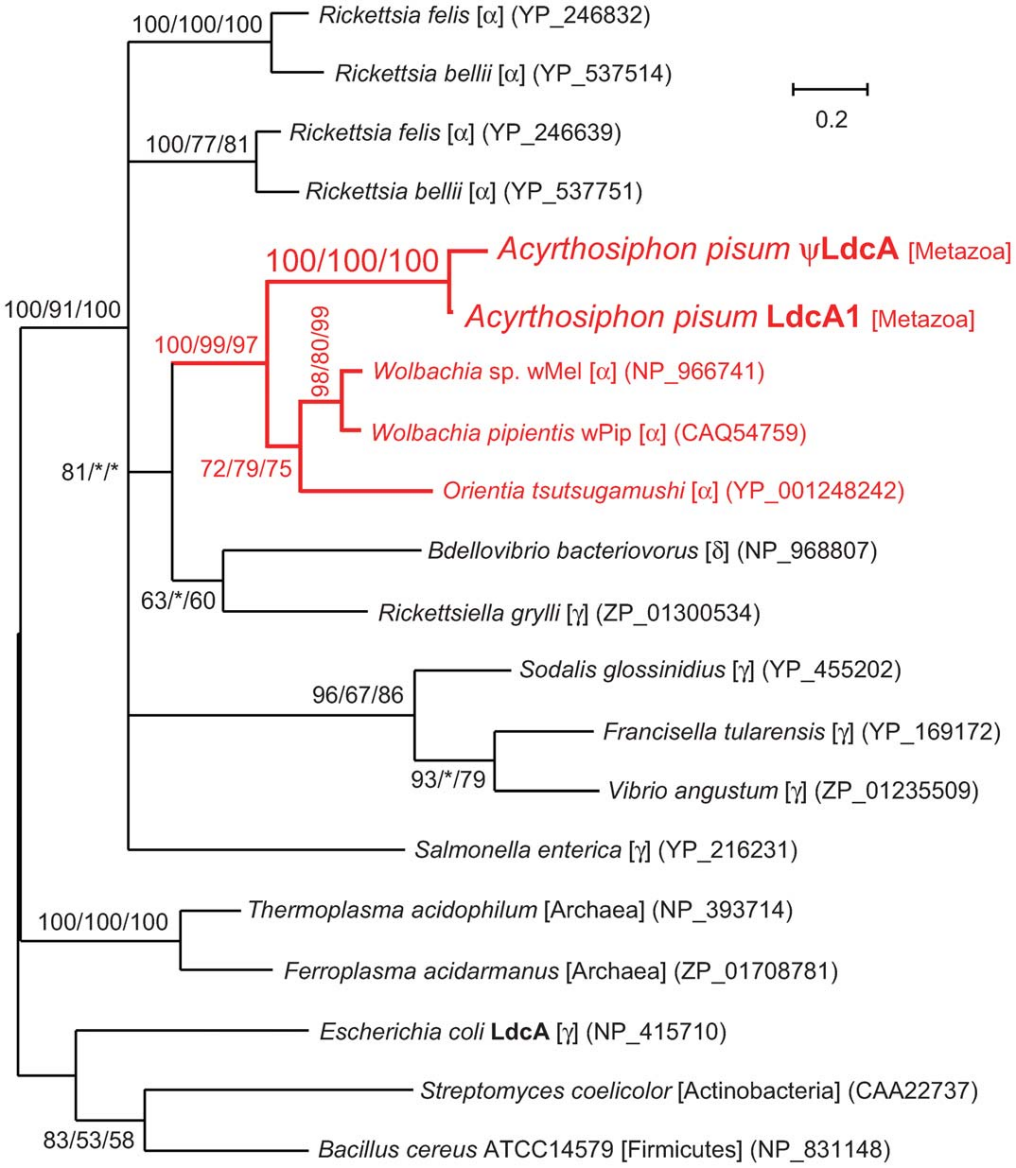
Phylogenetic position of the aphid LdcA proteins. A total of 136 aligned amino acid sites were subjected to the analysis. A Bayesian tree is shown; the ML tree and NJ tree exhibited substantially the same topologies. On each node, support values over 50 are shown (BI/ML/NJ). Asterisks (*) indicate support values lower than 50. Taxonomic positions (bacterial taxonomy unless otherwise stated) are shown in brackets.α, γ, and δ indicate proteobacterial classes. The *A. pisum*-*Rickettsiales* cluster is shown in red. Accessions of the sequences are shown in parentheses. Scale bar indicates substitutions per site.

Molecular phylogenetic analyses demonstrated that the *A. pisum LdcA1 and* ψ*LdcA* form a monophyletic clade (100% support in BI, ML, and NJ) that is sister to the clade of *ldcA*s of rickettsial bacteria, including *Wolbachia* (Alphaproteobacteria, Rickettsiales) (NP_966741) and *Orientia tsutsugamushi* (Alphaproteobacteria, Rickettsiales) (YP_001248242) (100% in BI; 99% in ML; 97% in NJ) (Figure 6). This branching pattern can be most simply explained by the hypothesis that an *ldcA* copy was transferred from *Wolbachia* or some other rickettsial bacterium to the aphid genome, followed by duplication, and subsequent inactivation of one copy. Symbionts from Rickettsiales are observed in some aphids [38],[40],[41], suggesting this bacterial clade as the source of this gene. However, the phylogeny is consistent with horizontal transfer among bacterial groups (Figure 6), and the *A*. *pisum* fragment potentially derives from another source such as a group of bacteria not yet sequenced. Mitochondria are also derived from the Alphaproteobacteria, but they can be ruled out as likely sources of this gene, since all animal mitochondria are extremely reduced in gene content and lack homologs of *ldcA*.

The remaining *LdcA* gene (*LdcA2* in the SCAFFOLD1029) found in this study (Table 1) contained a large sequence gap, and only 108 nucleotides of its potential protein-coding sequence had been determined. This 108 bp region of *LdcA2* was 100% identical to the corresponding region of *LdcA1* (Figure 5). Moreover, the BLASTN analysis using bl2seq revealed that an approximately 10-kb region containing *LdcA2* (total length unknown) in the SCAFFOLD1029 (45066 bp) is 97% identical to a region containing *LdcA1* (1364 bp) in the SCAFFOLD6884 (19038 bp). Regarding the SCAFFOLD11510 containing ψ*LdcA*, significant similarities to SCAFFOLD6884 and SCAFFOLD1029 were detected only in the ψ*LdcA* region. This may suggest that *LdcA2* also arose from a duplication event, and that its evolutionary history is relatively short in comparison to that of ψ*LdcA*. However, we cannot exclude the possibility that *LdcA1* and *LdcA2* are alleles of a single gene, as the sequenced aphid genomic sample was heterozygous for some genomic regions (IAGC, paper under review).

### Aphids appear to have acquired *AmiD* from a rickettsial bacterium

**Figure 7:**
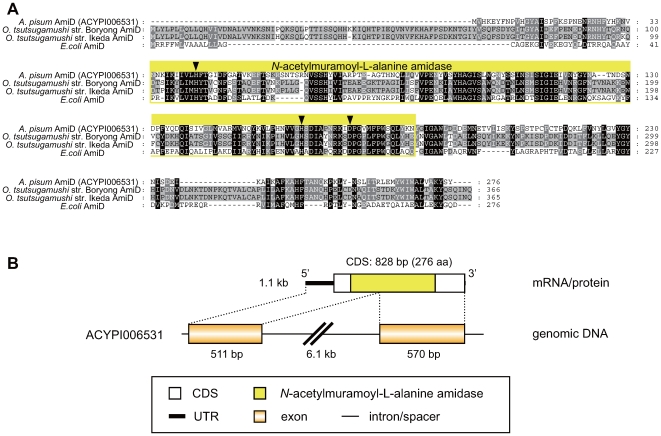
Structure of the aphid AmiD. (A) Alignment of amino acid sequences of AmiDs. Residues conserved in all lineages, three lineages, and two lineages are shaded black, dark gray, and light gray, respectively. Residues contributing to the domain structures are boxed. Arrowheads indicate three conserved amino acid residues involved in chelation of the zinc ion (His-34, His-154, and Asp-164) [43],[44]. Dashes (-) indicate alignment gaps. (B) Domain structure of the aphid AmiD protein and structures of the corresponding mRNA and genomic DNA.

Another LGT candidate, ACYPI006531 in the SCAFFOLD15270 (EQ126042), was similar to bacterial genes encoding *N*-acetylmuramoyl-L-alanine amidase (AmiD) (Table 1, Figure 7). This enzyme is also required for recycling murein (peptidoglycan), a component of the bacterial cell wall [42]. The top BLASTP hit for the predicted gene (XP_001945574.1) of ACYPI006531 was a putative *N*-acetylmuramoyl-L-alanine amidase [*O. tsutsugamushi* (Alphaproteobacteria, Rickettsiales)] (YP_001248113) (E = 1×10^−55^). Subordinate hits were either orthologs of AmiD or AmpD, two types *N*-acetylmuramoyl-L-alanine amidases that are characterized in *E. coli* [42]. The *A. pisum* gene ACYPI006531 was named *AmiD*, as it showed higher similarity to orthologs of AmiD than to AmpD. Moreover, as is the case for other AmiD orthologs, the *A. pisum* AmiD has an extra C-terminal tail (∼100 amino acids) that is absent from AmpD orthologs. This structural feature typifies AmiD, although the function of the tail is not known [42]. The amino acid sequence of *A. pisum* AmiD was 47% and 41% identical to AmiD proteins of *O. tsutsugamushi* and *E. coli*, respectively (Figure 7A). All three amino acids in the zinc-binding triad of AmiD (His-34, His-154, and Asp-164), which are essential for its catalytic activity [42]–[44], were conserved in the *A. pisum* ortholog. Figure 7B shows the structure of the aphid *AmiD* gene. The gene appeared to consist of 2 exons and a long single intron, although the intron contained two gaps.

**Figure 8:**
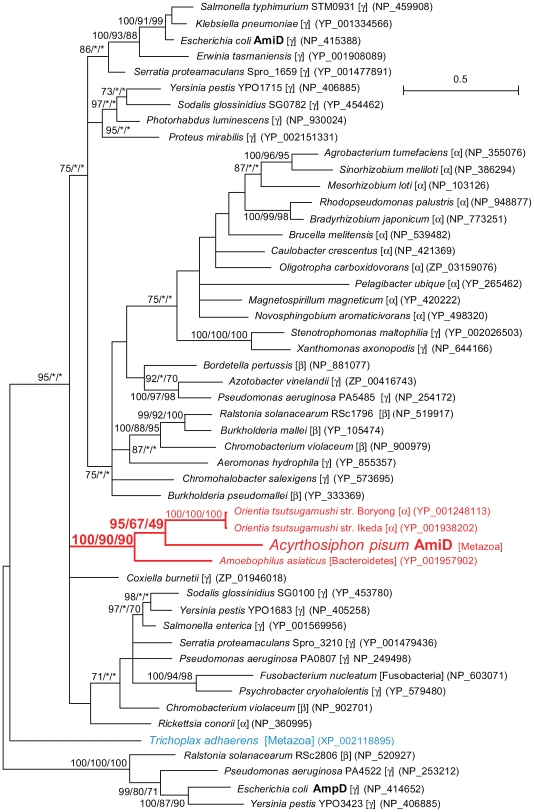
Phylogenetic position of the aphid AmiD protein. A total of 124 aligned amino acid sites were subjected to the analysis. A Bayesian tree is shown; the ML tree and NJ tree exhibited substantially the same topologies. On each node, support values over 50 are shown (BI/ML/NJ). Asterisks (*) indicate support values lower than 50. Taxonomic positions (bacterial taxonomy unless otherwise stated) are shown in brackets.α, β, and γ indicate proteobacterial classes. The *A. pisum*-*Orientia-Amoebophilus* cluster is shown in red. The sequence from the placozoan *T. adhaerens* is shown in blue. Accessions of the sequences are shown in parentheses. Scale bar indicates substitutions per site.

Phylogenetic analyses showed that the *A. pisum* AmiD is closely related to orthologs from Proteobacteria (Figure 8). Moreover, there was robust support (100% in BI; 90% in ML; 90% in NJ) for *A. pisum* AmiD forming a monophyletic clade with orthologs from intracellular symbiotic bacteria such as *O. tsutsugamushi* (Alphaproteobacteria) (YP_001248113) and *Amoebophilus asiaticus* (Bacteroidetes) (YP_001957902). *O. tsutsugamushi* is an intracellular bacterium that infects arthropods and mammals [45], whereas *A. asiaticus* is an intracellular symbiont of a unicellular eukaryote, *Acanthamoeba* [46]. This branching pattern can be most simply explained by the hypothesis that the aphid acquired *amiD* via LGT from a rickettsial bacterium. It is possible that *A. asiaticus* acquired *amiD* via LGT from a bacterium belonging to Proteobacteria, as the *A. asiaticus amiD* is distantly related to orthologs from other sequenced species of Bacteroidetes, and LGT is common among prokaryotes generally [47]. A putative ortholog of AmiD/AmpD was also detected in another metazoan species, the placozoan *Trichoplax adhaerens*. However, the phylogenetic tree showed that the *T. adhaerens* ortholog is distantly related to the aphid AmiD (Figure 8), implying that the ancestors of *A. pisum* and *T. adhaerens* independently acquired the genes from different lineages of bacteria.

### Aphid bLys is a fusion of a eukaryotic peptidase and a bacterial lysozyme

**Figure 9:**
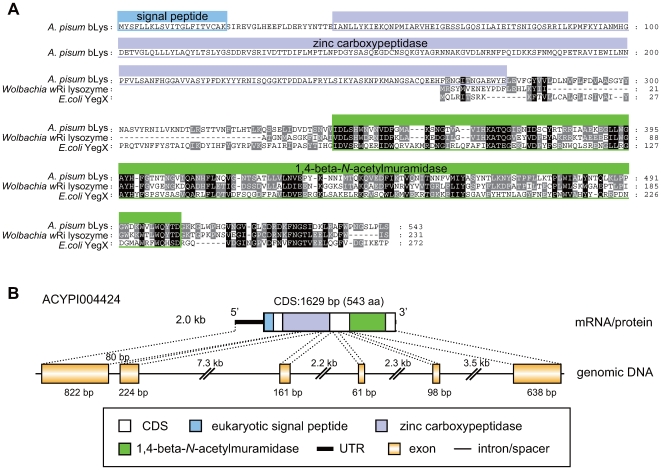
Structure of the aphid bLys. (A) Alignment of amino acid sequences of bLys orthologs. Residues conserved in all lineages and two lineages are shaded black and gray, respectively. Residues contributing to the domain structures are boxed. Dashes (-) indicate alignment gaps. (B) Domain structure of the aphid bLys protein and structures of the corresponding mRNA and genomic DNA.

ACYPI004424 in the SCAFFOLD2508 (EQ113280) appeared to encode a chimeric protein that consists of eukaryotic carboxypeptidase and a bacterial lysozyme (1,4-beta-*N*-acetylmuramidase) (Table 1, Figure 9). A conserved domain search at the NCBI website revealed that the carboxypeptidase (pfam00246, E = 3×10^−45^) and 1,4-beta-*N*-acetylmuramidase (pfam01183, E = 2×10^−22^) are encoded in its N-terminal region and C-terminal region, respectively. RT-PCR cloning of the transcript verified that the gene is transcribed and is truly chimeric (AB509281). The top hit of BLASTP for the C-terminal domain of the predicted gene model was 1,4-beta-*N*-acetylmuramidase [*Wolbachia* endosymbiont of *Drosophila simulans* (Alphaproteobacteria, Rickettsiales)] (YP_002727734) (E = 3×10^−54^). The subordinate hits were lysozymes of various lineages of bacteria. As these bacterial lysozyme genes lack common gene symbols, ACYPI004424 was tentatively named *bLys* (bacterial Lysozyme).

Lysozymes represent a family of enzymes that degrade bacterial cell walls by hydrolyzing the 1,4-beta-linkages between *N*-acetyl-D-glucosamine and *N*-acetylmuramic acid in murein heteropolymers [48]. They are ubiquitously distributed among living organisms and are believed to be essential for defense against bacterial infection. Lysozymes are classified into several types (*i.e.*, chicken, goose, invertebrate, plant, bacteria and phage types), and the *A. pisum* bLys was clearly categorized as a bacterial type (see below). Interestingly, unlike all other fully sequenced Metazoa, *A. pisum* appears to lack genes encoding canonical lysozymes [49]. If the bLys retains the bacteriolytic activity, this bacterium-derived lysozyme might compensate for the lack of canonical lysozymes in *A. pisum*. Figure 9B shows the chimeric structure of the aphid *bLys* gene. Bacterial lysozyme was encoded in the last (6th) exon, whereas eukaryotic carboxypeptidase was encoded in the 1st-3rd exons. The 1st exon also encoded a eukaryotic signal peptide, suggesting that the product is a secretory protein, as are other lysozymes.

**Figure 10:**
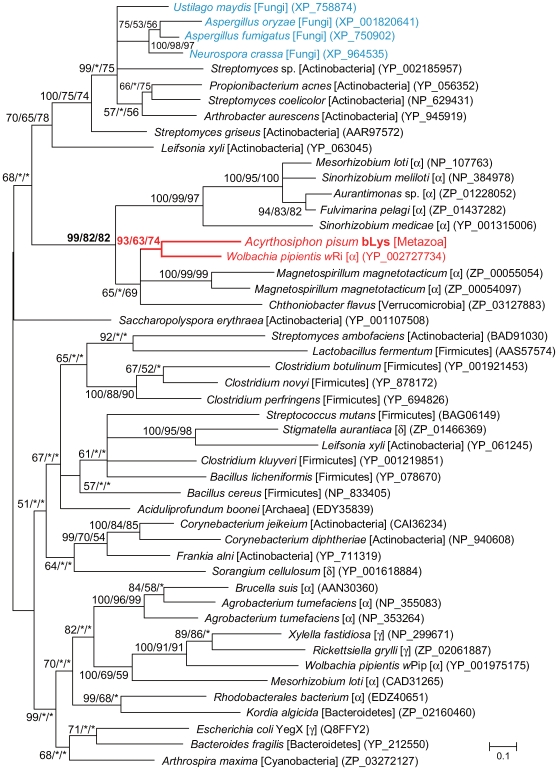
Phylogenetic position of the aphid bLys protein. A total of 133 aligned amino acid sites were subjected to the analysis. A Bayesian tree is shown; the ML tree and NJ tree exhibited substantially the same topologies. On each node, support values over 50 are shown (BI/ML/NJ). Asterisks (*) indicate support values lower than 50. Taxonomic positions (bacterial taxonomy unless otherwise stated) are shown in brackets.α, γ, and δ indicate proteobacterial classes. The *A. pisum*-*Wolbachia* cluster is shown in red. Sequences from the fungi are shown in blue. Accessions of the sequences are shown in parentheses. Scale bar indicates substitutions per site.

The amino acid sequence of the lysozyme domain of the *A. pisum* bLys was subjected to molecular phylogenetic analysis (Figure 10). The tree demonstrated that the aphid gene forms a clade with orthologs from Alphaproteobacteria (99% in BI; 82% in ML; 82% in NJ), and is especially closely related to a gene of *Wolbachia pipientis w*Ri (YP_002727734) (93% in BI, 63% in ML, 74% in NJ). This is consistent with the hypothesis that the *A. pisum bLys* was transferred from a *Wolbachia*-like rickettsial bacterium to an ancestral aphid genome.

### Aphid *RlpA* was duplicated after LGT

**Figure 11:**
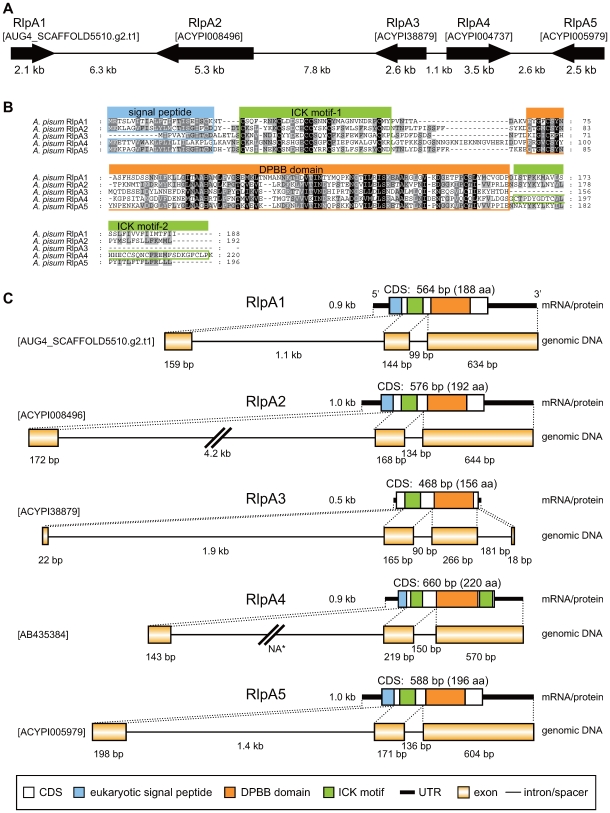
Structure of the aphid RlpAs. (A) Order and directions of *RlpA* genes in the SCAFFOLD5509. (B) Alignment of amino acid sequences of RlpAs. Residues conserved in all lineages, four lineages, and three lineages are shaded black, dark gray, and light gray, respectively. Dashes (-) indicate alignment gaps. Residues contributing to the domain structures are boxed. (C) Domain structures of the aphid RlpA proteins and structures of the corresponding mRNAs and genomic DNA sequences. *The length of the putative first intron of *RlpA4* is unknown, as the putative first exon identified by cDNA cloning (AB435384) was not found in the SCAFFOLD5509. This would be due to sequence gaps in this scaffold.

Five candidates (AUG4_SCAFFOLD5510.g2.t1, ACYPI008496, ACYPI38879, ACYPI004737, and ACYPI005979) appeared to encode bacterial rare lipoprotein A (RlpA) (Table 1). In contrast to the case of *LdcA*s, all of the five *RlpA* genes were clustered in a single scaffold, SCAFFOLD5509 (EQ116281) (Figure 11A). They were numbered consecutively following their order in the scaffold, and the *RlpA* gene that we reported previously (corresponding to ACYPI004737) [29],[30] was renamed *RlpA4*. The N-terminus of the computationally predicted gene model of ACYPI38879 was slightly different from what we reported previously (AB435384, AB435385) [30]. As our original predictions were based on full-length cDNA sequences and are highly reliable, we used these gene boundaries in the subsequent analyses. A double-ψ β-barrel (DPBB) domain that is conserved in bacterial RlpAs was conserved in all of the *A. pisum* RlpAs (encoded in the 3rd exon). In addition, an aphid-specific inhibitor cysteine-knot (ICK) domain, which is conserved in RlpA4 orthologs of three different aphid species [30], was observed at the N-terminal side of the DPBB domain of all the five *A. pisum* RlpAs (encoded in the 2nd exon) (Figure 11B and 11C). Signal peptides were detected in RlpA1, RlpA2, RlpA4, and RlpA5 (encoded in the 1st exon).

**Figure 12:**
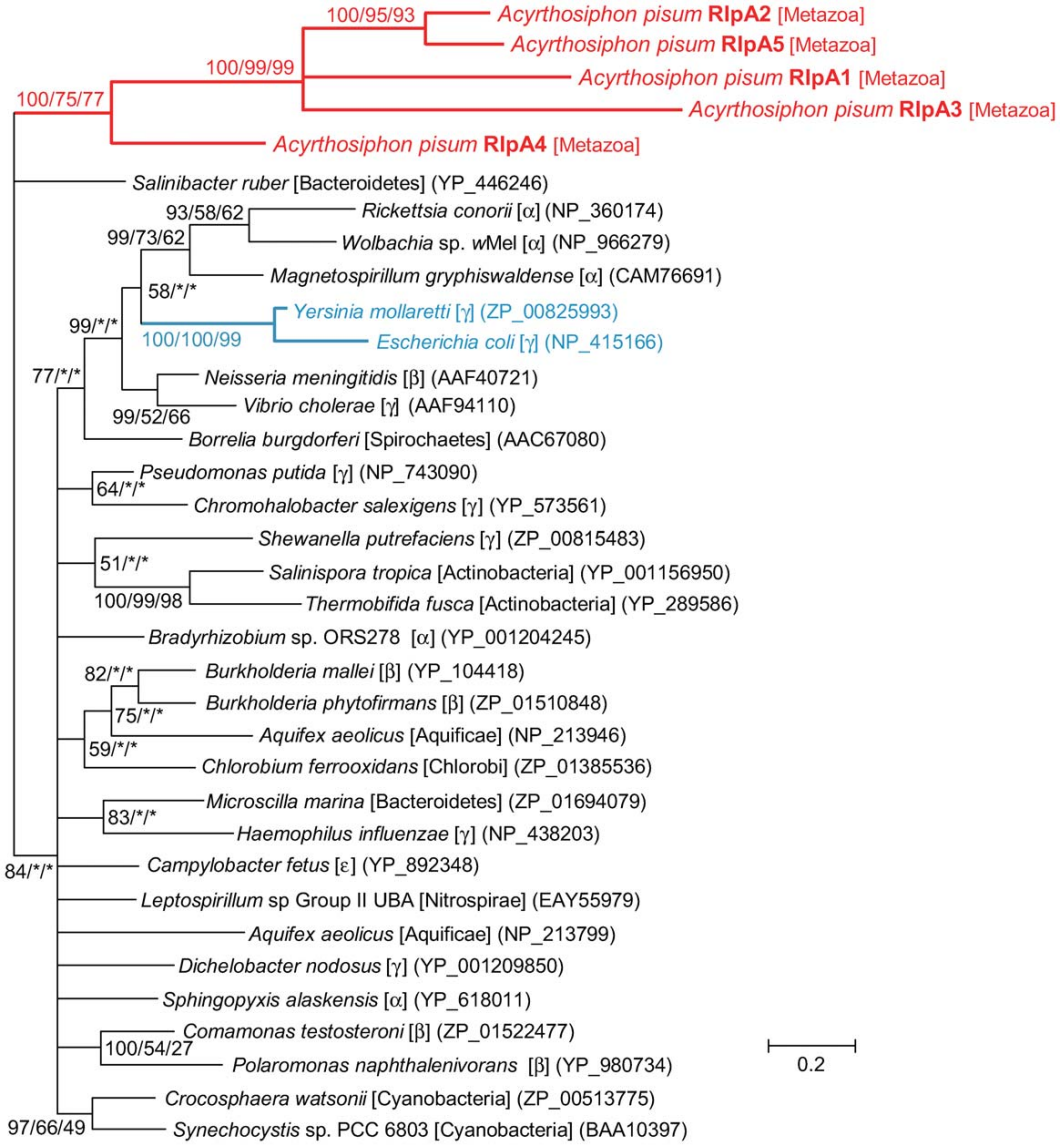
Phylogenetic position of the aphid RlpA proteins. A total of 88 aligned amino acid sites were subjected to the analysis. A Bayesian tree is shown; the ML tree and NJ tree exhibited substantially the same topologies. On each node, support values over 50 are shown (BI/ML/NJ). Asterisks (*) indicate support values lower than 50. Taxonomic positions (bacterial taxonomy unless otherwise stated) are shown in brackets.α, β, γ, and ε classes indicate proteobacterial classes. Accessions of the sequences are shown in parentheses. Scale bar indicates substitutions per site. The aphid clade and Enterobacteriaceae clade are shown in red and blue, respectively.

The molecular phylogenetic analysis indicated that the four newly found RlpAs (RlpA1, RlpA2, RlpA3, and RlpA5) are clustered with RlpA4 (100% in BI, 75% in ML, 77% in NJ) (Figure 12), which was previously demonstrated to be transferred from a bacterium to the aphid genome [30]. However, the phylogenetic positions of the aphid RlpAs were not clearly resolved within bacterial lineages. We tested the reliability in tree selection (approximately unbiased (AU) test [50]) and found that the possibility that the aphid clade clusters with the Enterobacteriaceae is quite low (p = 0.10); whereas the possibility that it exhibits the topology of the tree shown in Figure 12 was high (p = 0.91). Thus, it is unlikely that aphids acquired *RlpA* genes from ancestral *Buchnera*, as *Buchnera* branches with the family Enterobacteriaceae within the Gammaproteobacteria [16]. However, the tree of Figure 12 is suggestive of low resolution of the tree and/or of a history of horizontal transfer of *rlpA* among major bacterial groups, making the origin of the *A. pisum* copy unclear.

Their close localization in the genome, conserved exon/intron structures, and close phylogenetic positions suggest that the *A. pisum RlpA*s were duplicated after a single LGT from a bacterium. To further assess this possibility, we reanalyzed phylogenetic positions of aphid *RlpAs*, incorporating amino acid sequences of putative RlpA homologs of two other aphid species, *Myzus persicae* and *Aphis gossypii* (Figure S5). The tree showed that this gene was transferred before the divergence of Aphidini (containing *A. gossypii*) and Macrosiphini (containing *A pisum* and *M. persicae*), approximately 50–70 million years ago. This indicates that our strategies are effective in detecting rather ancient LGT.

### *LdcA1*, *AmiD*, and *RlpA*s are highly expressed in the bacteriocyte

**Figure 13:**
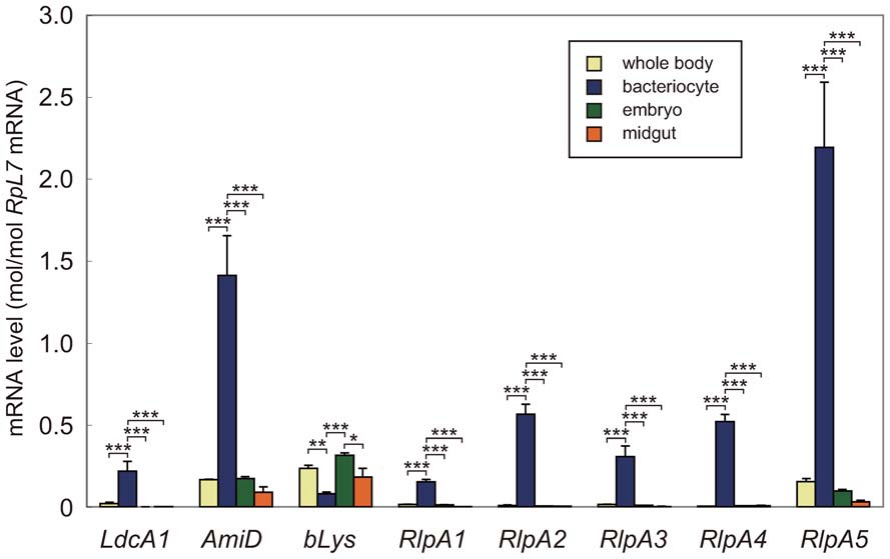
Expression profiles of LGT candidates. Ivory, blue, green, and orange columns represent expression levels in the whole body, the bacteriocyte, the embryo, and the midgut, respectively; bars, standard errors (*n* = 6). The expression levels are shown in terms of mRNA copies of the target genes per copy of mRNA for RpL7. Asterisks indicate statistically significant differences (Tukey-Kramer test; *, *p*<0.05; **, *p*<0.01; ***, *p*<0.001).

To examine the expression profiles of the aphid genes acquired from bacteria, we quantified their transcripts in the whole body, the bacteriocyte, the embryo, and the midgut, using real-time quantitative RT-PCR (Figure 13). Only one of the three copies of *LdcA* (*LdcA1*) was used for the analysis. Pilot RT-PCR experiments failed to amplify transcripts of ψ*DnaE* and ψ*AtpH*, whereas PCR successfully amplified genomic sequences of both (Figure 2), verifying the effectiveness of the primer sets. Failure of the RT-PCR amplification suggests that these loci are not transcribed at a significant level, further supporting the hypothesis that ψ*DnaE* and ψ*AtpH* are pseudogenes. RT-PCR successfully amplified transcripts of *LdcA1*, *AmiD*, *bLys, RlpA1*, *RlpA2*, *RlpA3*, *RlpA4*, and *RlpA5*, suggesting they are transcribed and possibly functional.

The genes that were positive by RT-PCR analysis were further characterized using quantitative RT-PCR to measure the level of their transcription. These experiments showed that expression of *LdcA1*, *AmiD*, *RlpA1-5* is highly upregulated in bacteriocytes. Transcripts for *LdcA1*, *AmiD*, *RlpA1*, *RlpA2*, *RlpA3*, *RlpA4*, and *RlpA5* were 11.6, 8.53, 10.2, 64.6, 22.3, 154, and 14.1-fold more abundant in the bacteriocyte than in the whole body, respectively (*p*<0.001, one-way ANOVA followed by Tukey-Kramer test) (Figure 13). For these genes, the transcript levels were invariably higher in the bacteriocytes than in the embryo and the midgut as well (*p*<0.001, Tukey-Kramer test). In contrast, the transcript for *bLys* appeared to be less abundant in the bacteriocyte than in other organs. The level of the *bLys* transcript in the bacteriocyte was only 33.7% of that in the whole body (*p*<0.01, Tukey-Kramer test), whereas the levels of the *bLys* transcript in the embryo and the midgut were comparable to that in the whole body (*p*>0.05, Tukey-Kramer test).

## Discussion

In this study, using a number of different approaches, we identified 12 genes or gene fragments that are highly likely to have been transferred from bacteria to the genome of an ancestor of *A. pisum*. Unexpectedly, however, we found no functional genes that seem to have been transferred from *Buchnera* to the aphid genome. Only two pseudogenes, ψ*DnaE* and ψ*AtpH*, were shown to be of *Buchnera* origin, and both are highly truncated and appear not to be transcribed. Although a number of gaps still remain in the genome assembly of *A. pisum* (Acyr_1.0), exhaustive analyses of gene inventory and transcriptome data suggest that few genes are hidden in such gaps [51], indicating that the assembly is nearly complete (IAGC, under review). We do not exclude the possibility that we have missed a very limited number of LGT candidates, but that would not much affect the overall result. Moreover, results for RlpA suggest that rather ancient transfers could be detected using our search strategies (Figure S5). Thus, the present results rule out the hypothesis that the reductive evolution of the *Buchnera* genome has been enabled by LGT to the host aphid genome.

The lack of LGT from *Buchnera* demonstrates a clear difference between *Buchnera* and organelles such as mitochondria and plastids, which have transferred a number of essential genes into the host chromosome [7],[8]. In *Buchnera*, loss of genes known to be essential for model organisms, such as *E. coli*, might reflect the coadaptation of other *Buchnera* genes, which may evolve additional functions, or coadaptation of the host, which may evolve to support its mutualistic symbiont [29],[52].

Besides the two pseudogenes apparently derived from *Buchnera*, the 10 LGT candidates were three genes of LD-carboxypeptidase (*LdcA*), five genes of rare lipoprotein A (*RlpA*), and a single gene each of *N*-acetylmuramoyl-L-alanine amidase (*AmiD*), and 1,4-beta-*N*-acetylmuramidase (*bLys*). One each of *LdcA*s (*LdcA1*) and *RlpA*s (*RlpA4*) were originally found in our previous studies [29],[30]. Phylogenetic analyses suggested that *LdcA*s, *AmiD*, and *bLys* were derived from rickettsial bacteria closely related to the extant *Wolbachia* spp. (Alphaproteobacteria, Rickettsiales) and *Orientia tsutsugamushi* (Alphaproteobacteria, Rickettsiales), both of which are intracellular symbionts of Metazoa. *Wolbachia* are found in various lineages of arthropods and nematodes [16], [22]-[27],[41],[53], whereas *Orientia* infect arthropods and mammals [45]. Although the LSR1 strain used for the genome sequencing lacks rickettsial symbionts, infections of *Wolbachia* and *Rickettsia* are sporadically observed in aphids [38],[40],[41]. Thus, a previous infection may have been the source of these transferred genes [30]. Recent studies have revealed that genomes of various animal lineages have DNA sequences that appear to have been transferred from *Wolbachia* [22]–[27],[30]. Dunning Hotopp *et al*. screened the genomes of a wide variety of nematodes and arthropods, including *A. pisum*, for *Wolbachia*-like sequences [24]. However, they did not detect any of the five *Wolbachia*-like genes identified in the present study. This appears to reflect their use of a higher threshold (>80% nucleotide identity), using only extant *Wolbachia* genomes as queries, aiming to detect recent LGTs from *Wolbachia*. In contrast, we conducted exhaustive analyses to identify all possible (ancient and recent) LGTs from all possible bacterial lineages, resulting in identification of 12 promising candidates. This implies that more extensive searches of other animal genomes could reveal many more LGT candidates. Thus, the *A. pisum* genome may not be unusual in containing genes acquired from bacteria.

Rickettsial bacteria invade various types of host cells [13],[16],[38],[41], and in some cases are concentrated in germ cells [53]. In contrast, during most of their life stages, *Buchnera* are confined within bacteriocytes, which are somatic cells that are segregated from germ cells [13]–[16]. *Buchnera* cells are freed from the maternal bacteriocytes and are localized in the host germ line only when being transmitted to the next generation [13],[54],[55]. LGT must take place in the germ line for a transferred gene to be inherited across generations, and the difference between rickettsial bacteria and *Buchnera* in proximity to nuclei of germ cells may affect their rates of DNA transfer to host genomes. In this context, nuclear mitochondrial-like sequences (numts) may give some insight. As mitochondria reside in essentially all types of host cells including germ line cells, the nuclear genome has frequent opportunity to contact and acquire genomic fragments from mitochondria. Indeed, a total of 56 pseudogene sequences derived from transferred mitochondrial DNA was detected in the nuclear genome of *A. pisum*; these were estimated to represent approximately 35 transfer events, taking into account that some DNA transfers involved fragments bearing more than one gene and some DNA transfers were followed by subsequent duplication (IAGC, paper under review). Thus, detectable DNA transfers from mitochondria were more frequent than LGTs from bacteria, consistent with the hypothesis that proximity to germ line nuclei facilitates DNA transfer.

Interestingly, all of the *A. pisum* genes of apparent rickettsial origin (*LdcA*s, *AmiD*, and *bLys*) encoded enzymes for metabolism of murein (peptidoglycan), a component of the bacterial cell wall. In bacteria, LdcA and AmiD are required for recycling murein [42], whereas lysozymes are utilized to hydrolyze it [48]. Quantitative RT-PCR demonstrated that expression of the *A. pisum LdcA1* and *AmiD* were highly upregulated in the bacteriocyte, which is the cell that harbours *Buchnera*. As *Buchnera* possesses a cell wall composed of murein [56], but lacks both *ldcA* and *amiD* [9], it is plausible that the laterally transferred *LdcAs* and *AmiD* in the *A. pisum* bacteriocyte may have compensatory functions to support the survival of *Buchnera*, as proposed previously for *LdcA1* [30]. While neither function nor phylogenetic position of the aphid *RlpA*s is yet known, their proximity in the genome, conserved exon/intron structures, and close phylogenetic positions suggested that the five *RlpA* genes of *A. pisum* were generated from a single gene, by duplications following LGT. Moreover, the expression of all five genes was shown to be upregulated in the bacteriocyte, implying that all of the duplicated *RlpA*s function in the maintenance of *Buchnera.* Gene duplications after LGT were also shown for *LdcA* genes.

In contrast to the case of *LdcA1* and *AmiD*, the transcript for *bLys* was more abundant in other organs than in the bacteriocyte. In this context, it is notable that, unlike all other fully sequenced Metazoa, *A. pisum* lacks genes encoding canonical lysozymes [49]. Lysozymes are ubiquitously distributed among living organisms and are believed to be essential for defense against bacterial infection [48]. Retention of bacteriolytic activity by bLys might compensate for the lack of canonical lysozymes in *A. pisum*. The relatively high level of expression of *bLys* in the whole body of the pea aphid, observed in our study, might reflect a role of the bLys protein protecting the aphid's body from infectious bacteria. Conversely, high expression of *bLys* in the bacteriocyte might have a detrimental effect on the *Buchnera* cell wall, and the fact that it is not highly expressed in that tissue is consistent with *Buchnera*'s importance in aphid nutrition [15], [18]–[21].

A previous study on two LGT candidates (*LdcA1* and *RlpA4*) suggested that apparent functional genes laterally transferred from bacteria have acquired some eukaryotic features [30]. This observation was further supported by the present, more comprehensive analyses. Spliceosomal-type introns were found in all the genes that were transcribed (*LdcA1*, *LdcA2*, *AmiD*, *bLys*, and *RlpA1-5*). This type of intron has not been observed in bacterial genes, indicating that these genes acquired introns after they were transferred to the aphid nuclear genome. Moreover, RlpAs and bLys display chimeric structures that consist of eukaryotic domains and prokaryotic domains. As the boundaries of the eukaryotic and prokaryotic domains of these proteins were consistent with the locations of introns, the chimeric structures of these genes might have come into being as the result of exon-shuffling [30],[57].

In addition to providing strong evidence that *A. pisum* did not acquire functional genes from *Buchnera*, this study also provided evidence that 1) DNA fragments can be transferred from mutualistic intracellular bacteria to the nuclear genome of metazoan hosts; 2) Genes transferred from bacteria can function in the recipient Metazoa; 3) Transferred genes may be utilized for facilitating mutualistic associations between Metazoa and symbiotic bacteria.

Although we can rule out the hypothesis that *Buchnera* genome reduction was dependent on transfer of genes to its host, it remains possible that other mutualistic intracellular bacteria have transferred genes to genomes of their metazoan hosts, as in mitochondria and plastids [7],[8]. Recently, several bacterial symbionts have been found to have genomes much smaller than that of *Buchnera*, and these symbionts are promising candidates for further investigation [1]–[4]. The most extreme cases are *Carsonella ruddii* str. Pv (Gammaproteobateria), the bacteriocyte symbiont of a psyllid, and *Hodgkinia cicadicola* (Alphaproteobacteria), a bacteriocyte symbiont of singing cicadas. The genomes of these two unrelated bacteria (NC_008512 and CP001226) are 160 kb and 144 kb in size, respectively, only a quarter of that of *Buchnera* str. APS (NC_002528), and they are lacking numerous genes considered essential for life [1],[2],[4].

## Materials and Methods

### Complete genome sequence and predicted gene models of *A. pisum*

The first release of the genome assembly of the pea aphid, *Acyrthosiphon pisum*, (Acyr_1.0) was obtained from the Human Genome Sequencing Center at Baylor College of Medicine (http://www.hgsc.bcm.tmc.edu/projects/aphid/). Predicted gene models (Gnomon, RefSeq, etc.) of *A. pisum* were obtained from the National Center for Biotechnology Information (NCBI; http://www.ncbi.nlm.nih.gov/Ftp/).

### Screening of unassembled reads

90,678 reads that were precluded from the *A. pisum* genome assembly (Acyr_1.0) were retrieved from the Human Genome Sequencing Center at the Baylor College of Medicine (http://ftp.peaaphidgenome.hgsc.bcm.tmc.edu/peaaphidftp.html). BLASTX and BLASTN similarity searches [58] were performed using the genome of *Buchnera aphidicola* str. APS (NC_002252, NC_002253, NC_002528) [9], custom bacterial databases, RefSeq invertebrate databases, NCBI non-redundant (nr) databases, and the *A. pisum* genome assembly (Acyr_1.0). The bacterial nucleotide/protein databases were constructed using complete genome sequences of 35 representative bacterial species belonging to Proteobacteria or Firmicutes (8, 21, 3, and 3 species from Alphaproteobacteria, Gammaproteobacteria, Betaproteobacteria, and Firmicutes, respectively) (Table S1). The assembly of the discarded reads were performed with the phred/phrap package [59] using default parameters.

### BLASTP–based screening of the *A. pisum* genome for laterally transferred genes

In this study, we define “open reading frames (ORFs)” as potential protein-coding sequences regardless of the presence/absence of initiation codons; namely, DNA stretches that begin with the first nucleotide after the previous stop codon and ends with a stop codon, with no stop codons in between. All ORFs having an inferred polypeptide length of at least 60 amino acids were extracted from the genome assembly of *A. pisum* (Acyr_1.0). Overlaps of ORFs encoded in different reading frames were permitted. The algorithm for the ORF extraction did not include prediction of exon/intron boundaries. Potential polypeptides (PPPs) were deduced from these ORFs and were used for analyses. BLASTP similarity searches [58] were conducted against the bacterial protein database and the invertebrate protein database. The invertebrate protein database was retrieved from NCBI (Reference Sequence Release 30). BLASTP searches against the non-redundant (nr) protein database were conducted at the NCBI website.

### BLASTX–based screening of the *A. pisum* genome for laterally transferred genes

The genome assembly of *A. pisum* (Acyr_1.0) was divided into lengths of 1,000 nucleotides (nts), overlapping by 200 nts. BLASTX [58] similarity searches were performed using the NCBI nr protein database (-e 1e-2 -F “m S”) and a custom database (-e 1 -F “m S”) containing proteomes of 714 prokaryotic species (Table S2), along with the proteomes from the insects *Drosophila melanogaster*, *Anopheles gambiae*, *Apis mellifera*, and *Tribolium castaneum* (Figure S2.2). See also Text S1.

### BLASTN screens with aphid endosymbiont genomes

BLASTN searches were performed using the *Buchnera* str. APS genome (NC_002252, NC_002253, NC_002528) and the *Hamiltonella defensa* 5AT genome (ND_12751, ND_12752) as queries on the *A. pisum* genomic database. These searches have the potential to reveal recently transferred sequences that do not fall within protein-coding genes. The requirement for significance was E value < e-05. For significant hits, the *A. pisum* sequences from alignments were used as queries in BLASTN searches on nr nucleotide database at NCBI. Sequences were eliminated as LGT candidates if top hits in these searches were from other insect genomes. Scaffolds with hits in the initial searches were also checked against the list of scaffolds previously designated as bacterial contaminants. Scaffolds were eliminated as LGT candidates if included on this list or if the whole scaffold was both <2 kb in length and lacked any significant hits to another animal genome.

### Real-time quantitative PCR

Two *A. pisum* strains free from secondary symbionts were used for the analysis. One was strain LSR1, the North American strain that was used for the genome sequencing, and the other was strain ISO, the Japanese strain that was used for our previous transcriptome analysis of bacteriocytes [29]. DNA was extracted from three and four different batches of LSR1 and ISO, respectively. Quantification was performed with the LightCycler instrument and FastStart DNA Master^PLUS^ SYBR Green I kit (Roche), as described previously [29]. The primers used are listed in Table 2. The running parameters were: 95°C for 10 min, followed by 35 cycles of 95°C for 10 s, 55°C for 5 s, and 72°C for the time shown in Table 2. Results were analyzed using LightCycler software version 3.5 (Roche), and the copy numbers were normalized to that of a ribosomal protein gene, *RpL7*. Statistical analyses were performed using one-way ANOVA and the Tukey-Kramer test.

**Table 2:** Primer sets used for quantitative PCR/RT–PCR.

Target	Forward primer 5′-3′	Reverse primer 5′-3′	Amplicon size (bp)	Extension time (s)
*LdcA1*	CAACCTGACGCTAGTCGAGAACT	CACGTCCTCCAAGAACACGAT	82	4
*AmiD*	GGGGCAACTACTCGTCAATC	ACGGGTCCCATGAATCATTAG	90	4
*bLys*	TGCAACAACCAGAAACCAGAGC	ACAACAGCACCACCATGAAAATTTGC	88	4
*RlpA1*	GCTATGTTGCCAGTTGGCTCAG	GGTTCCGTTCTTTGGTTGCATATAGG	102	5
*RlpA2*	GTTGTCCAGAGAAACAGCCAAGGT	GAATTCTCCAAACGGGGTACAAC	87	4
*RlpA3*	GAAGGACGACAGTCATCACG	ACATCAGAGCAGCGTCATTGG	87	4
*RlpA4*	CGGCGGACGGTAAGGTAAT	ACTGTACCGGGCCTGTGTTC	81	4
*RlpA5*	CAATCCGGAAAATAAGGCAGTTGA	CACTTCAACTTTTGTGCCTGGTGG	97	4
ψ*DnaE*	GCTTCTACTCAAGAAGGATATAAC	GCTCTCGTGTAATTATGTACC	272	11
ψ*AtpH*	AATTGTTGGATTGAGATCAGCA	ACAAAGACTGTTCTCATATTGTTCG	80	4

### Structural analysis

Domain structures of predicted proteins were analyzed using the CD-search at the NCBI website [60]. Similarities between two sequences were analyzed by using the bl2seq tool at the NCBI website [58]. The presence and location of signal peptides were predicted by using the program SignalP 3.0 [61].

### Molecular phylogenetic analysis

Multiple alignment was performed using the program MAFFT 5.8 [62], followed by manual refinement. Aligned sites that included alignment gap(s) were omitted from the analysis. Molecular phylogenetic analyses were conducted by three methods, Bayesian inference (BI), maximum likelihood (ML), and neighbor joining (NJ). In the BI analysis, we used the program MrBayes 3.1.2 [63]. In total, 4,000–50,000 trees were obtained (ngen 400,000–5,000,000, samplefreq 100), and the first 2,000–40,000 of these were considered as “burn in” and discarded. We confirmed that the potential scale reduction factor (PSRF) was around 1.00 for all parameters and that the average standard deviation of split frequencies converged towards zero. A posterior probability of each node was used for the support value of the node. ML trees were estimated using the program RAxML Version 7.0.0 [64]. Bootstrap values were obtained by generating 1,000 bootstrap replications. NJ trees were constructed using the program Neighbor in PHYLIP 3.6 [65]. Pairwise distances were calculated by the program Tree-Puzzle 5.2 [66]. Bootstrap values were obtained by generating 1,000 bootstrap replications. We used the program ProtTest v1.4 [67] for the selection of the substitution models of amino acid sequences. The WAG + gamma + Inv model was used for the phylogenetic analysis except for that for DnaE. For the estimation of the DnaE tree, the Blossom62 + gamma model was used.

### Approximately unbiased test

To test whether the *A. pisum* RlpA might be derived from an ancestral *Buchnera* genome (and therefore closely related to the Enterobacteriaceae), an approximately unbiased (AU) test was conducted using the program package Treefinder version Oct. 2008 (distributed by the author at www.treefinder.de). For the analysis, the ML tree inferred by molecular phylogenetic analysis was used. The tree topology of the ML tree was fixed except for the phylogenetic position of the aphid RlpA cluster. All possible alternative positions of aphid RlpA cluster in the tree were analyzed, to assess the hypothesis that the aphid copy fell on the branch to Enterobacteriaceae.

### RT–PCR cloning of *bLys*

Strain ISO was used for the analysis. The insects were reared on *Vicia faba* at 15°C in a long-day regime of 16 hr light and 8 hr dark. RNA was isolated from the whole bodies of 12–15 day-old parthenogenetic apterous adults using TRIzol reagent, followed by RNase-free DNase I treatment. First-strand cDNAs were synthesized using pd(N)6 primer and PrimeScript reverse transcriptase (Takara). PCR primers were bLys_1F (5′- CCATTAGCTACTAATTGTCTAGTAAG -3′) and bLys_2004R (5′- TCATGAAAGAGGTAAACTTCCATTTG -3′). Running parameters were 94°C for 5 min, followed by 35 cycles of 94°C for 30 s, 57°C for 30 s, and 72°C for 2 min. The PCR product was cloned using pGEM-T easy vector system (Promega).

### Real-time quantitative RT–PCR

RNA was isolated from the whole bodies, bacteriocytes, embryos, and midguts of 12–15 day-old parthenogenetic apterous adults of the ISO strain as described above. First-strand cDNAs were synthesized and quantification was performed using the LightCycler system as described above. Primer sets and running parameters were the same as those used for the quantitative PCR except that the number of PCR cycles was 45. The relative expression levels were normalized to mRNA for the ribosomal protein RpL7. Statistical analyses were performed using one-way ANOVA and the Tukey-Kramer test.

## Supporting Information

Figure S1 Flow chart of the evaluation of the individual reads precluded from the genome assembly.
(0.03 MB PDF)

Figure S2 Flow chart of the BLASTX-based screening of the *A. pisum* genome for LGT candidates.
(0.04 MB PDF)

Figure S3 Alignment of amino acid sequences of DnaEs. Residues conserved in three and two lineages are shaded black and gray, respectively. Triangles and reverse-triangles indicate frameshift deletion and insertion, respectively, in the aphid ψ*DnaE*. Dashes (-) indicate alignment gaps. Asterisks (*) indicate gaps caused by frameshifts.
(0.49 MB PDF)

Figure S4 Alignment of amino acid sequences of AtpHs. Residues conserved in all and two lineages are shaded black and gray, respectively. Dashes (-) indicate alignment gaps. Dots (.) indicate stop codons.
(0.43 MB PDF)

Figure S5 Phylogenetic position of RlpA proteins from three aphid species. The legend is the same as for Figure 12. Circles indicate inferred splits of the ancestors of *A. pisum* and *Myzus persicae*. Rhombi indicate inferred duplications of RlpA. Amino acid sequences of RlpA proteins from *M. persicae* and *Aphis gossypii* were deduced from assembled EST sequences that were retrieved from NCBI. The accession numbers of the ESTs were DW014944, DW011752, ES221611, ES222724, and DW013043 for *M. persicae RlpA1*, EE571687 and EE264310 for *M. persicae RlpA3*, ES221157, EE263538, EE571585, EE262867, and ES220852 for *M. persicae RlpA5*, DR395894, DR393442, and DR391922 for *A. gossypii RlpA1*, and DR391796 for *A. gossypii RlpA4*.
(0.06 MB PDF)

Table S1 List of bacteria used to construct bacterial databases.
(0.03 MB DOC)

Table S2 List of bacteria and archaea used to construct a protein database (for the BLASTX-based screening).
(0.07 MB DOC)

Table S3 Scaffolds inferred to be of bacterial contaminants.
(0.06 MB XLS)

Text S1 Screening based on BLASTX.
(0.02 MB DOC)

Text S2 Relative rate test for the *A. pisum* ψ*DnaE*.
(0.03 MB DOC)

## References

[1] Nakabachi A, Yamashita A, Toh H, Ishikawa H, Dunbar HE (2006). The 160-kilobase genome of the bacterial endosymbiont *Carsonella*.. Science.

[2] Nakabachi A, Bourtzis K, Miller TA (2008). Mutualism revealed by symbiont genomics and bacteriocyte transcriptomics.. Insect Symbiosis.

[3] McCutcheon JP, Moran NA (2007). Parallel genomic evolution and metabolic interdependence in an ancient symbiosis.. Proc Natl Acad Sci U S A.

[4] McCutcheon JP, McDonald BR, Moran NA (2009). Origin of an alternative genetic code in the extremely small and GC-rich genome of a bacterial symbiont.. PLoS Genet.

[5] Andersson SG (2006). The bacterial world gets smaller.. Science.

[6] Koonin EV, Wolf YI (2008). Genomics of bacteria and archaea: the emerging dynamic view of the prokaryotic world.. Nucleic Acids Res.

[7] Dyall SD, Brown MT, Johnson PJ (2004). Ancient invasions: from endosymbionts to organelles.. Science.

[8] Poole AM, Penny D (2007). Evaluating hypotheses for the origin of eukaryotes.. Bioessays.

[9] Shigenobu S, Watanabe H, Hattori M, Sakaki Y, Ishikawa H (2000). Genome sequence of the endocellular bacterial symbiont of aphids *Buchnera* sp. APS.. Nature.

[10] Tamas I, Klasson L, Canback B, Naslund AK, Eriksson AS (2002). 50 million years of genomic stasis in endosymbiotic bacteria.. Science.

[11] van Ham RC, Kamerbeek J, Palacios C, Rausell C, Abascal F (2003). Reductive genome evolution in *Buchnera aphidicola*.. Proc Natl Acad Sci U S A.

[12] Perez-Brocal V, Gil R, Ramos S, Lamelas A, Postigo M (2006). A small microbial genome: the end of a long symbiotic relationship?. Science.

[13] Buchner P (1965). Endosymbiosis of animals with plant microorganisms..

[14] Munson MA, Baumann P, Kinsey MG (1991). *Buchnera* gen. nov. and *Buchnera aphidicola* sp. nov., a taxon consisting of the mycetocyte-associated, primary endosymbionts of aphids.. Int J Syst Bacteriol.

[15] Douglas AE (1998). Nutritional interactions in insect-microbial symbioses: aphids and their symbiotic bacteria *Buchnera*.. Annu Rev Entomol.

[16] Moran NA, McCutcheon JP, Nakabachi A (2008). Genomics and evolution of heritable bacterial symbionts.. Annu Rev Genet.

[17] Moran NA, Munson MA, Baumann P, Ishikawa H (1993). A molecular clock in endosymbiotic bacteria is calibrated using the insect hosts.. P Roy Soc Lond B Bio.

[18] Febvay G, Liadouze I, Guillaud J, Bonnot G (1995). Analysis of energetic amino acid metabolism in *Acyrthosiphon pisum*: a multidimensional approach to amino acid metabolism in aphids.. Arch Insect Biochem.

[19] Sasaki T, Ishikawa H (1995). Production of essential amino acids from glutamate by mycetocyte symbionts of the pea aphid, *Acyrthosiphon pisum*.. J Insect Physiol.

[20] Nakabachi A, Ishikawa H (1997). Differential display of mRNAs related to amino acid metabolism in the endosymbiotic system of aphids.. Insect Biochem Mol Biol.

[21] Nakabachi A, Ishikawa H (1999). Provision of riboflavin to the host aphid, *Acyrthosiphon pisum*, by endosymbiotic bacteria, *Buchnera*.. J Insect Physiol.

[22] Kondo N, Nikoh N, Ijichi N, Shimada M, Fukatsu T (2002). Genome fragment of *Wolbachia* endosymbiont transferred to X chromosome of host insect.. Proc Natl Acad Sci U S A.

[23] Fenn K, Conlon C, Jones M, Quail MA, Holroyd NE (2006). Phylogenetic relationships of the *Wolbachia* of nematodes and arthropods.. PLoS Pathog.

[24] Dunning Hotopp JC, Clark ME, Oliveira DC, Foster JM, Fischer P (2007). Widespread lateral gene transfer from intracellular bacteria to multicellular eukaryotes.. Science.

[25] Nikoh N, Tanaka K, Shibata F, Kondo N, Hizume M (2008). *Wolbachia* genome integrated in an insect chromosome: evolution and fate of laterally transferred endosymbiont genes.. Genome Res.

[26] Klasson L, Kambris Z, Cook PE, Walker T, Sinkins SP (2009). Horizontal gene transfer between *Wolbachia* and the mosquito *Aedes aegypti*.. BMC Genomics.

[27] Woolfit M, Iturbe-Ormaetxe I, McGraw EA, O'Neill SL (2009). An ancient horizontal gene transfer between mosquito and the endosymbiotic bacterium *Wolbachia pipientis*.. Mol Biol Evol.

[28] Aikawa T, Anbutsu H, Nikoh N, Kikuchi T, Shibata F (2009). Longicorn beetle that vectors pinewood nematode carries many *Wolbachia* genes on an autosome.. Proc Biol Sci.

[29] Nakabachi A, Shigenobu S, Sakazume N, Shiraki T, Hayashizaki Y (2005). Transcriptome analysis of the aphid bacteriocyte, the symbiotic host cell that harbors an endocellular mutualistic bacterium, *Buchnera*.. Proc Natl Acad Sci U S A.

[30] Nikoh N, Nakabachi A (2009). Aphids acquired symbiotic genes via lateral gene transfer.. BMC Biol.

[31] Nakabachi A, Ishikawa H, Kudo T (2003). Extraordinary proliferation of microorganisms in aposymbiotic pea aphids, *Acyrthosiphon pisum*.. J Invertebr Pathol.

[32] Lazzaro BP, Sceurman BK, Clark AG (2004). Genetic basis of natural variation in *D. melanogaster* antibacterial immunity.. Science.

[33] Grenier AM, Duport G, Pages S, Condemine G, Rahbe Y (2006). The phytopathogen *Dickeya dadantii* (*Erwinia chrysanthemi* 3937) is a pathogen of the pea aphid.. Appl Environ Microbiol.

[34] Degnan PH, Yu Y, Sisneros N, Wing RA, Moran NA (2009). *Hamiltonella defensa*, genome evolution of protective bacterial endosymbiont from pathogenic ancestors.. Proc Natl Acad Sci U S A.

[35] Blattner FR, Plunkett G, Bloch CA, Perna NT, Burland V (1997). The complete genome sequence of *Escherichia coli* K-12.. Science.

[36] Komaki K, Ishikawa H (2000). Genomic copy number of intracellular bacterial symbionts of aphids varies in response to developmental stage and morph of their host.. Insect Biochem Mol Biol.

[37] Moran NA, Degnan PH, Santos SR, Dunbar HE, Ochman H (2005). The players in a mutualistic symbiosis: insects, bacteria, viruses, and virulence genes.. Proc Natl Acad Sci U S A.

[38] Sakurai M, Koga R, Tsuchida T, Meng XY, Fukatsu T (2005). *Rickettsia* symbiont in the pea aphid *Acyrthosiphon pisum*: novel cellular tropism, effect on host fitness, and interaction with the essential symbiont *Buchnera*.. Appl Environ Microbiol.

[39] Templin MF, Ursinus A, Holtje JV (1999). A defect in cell wall recycling triggers autolysis during the stationary growth phase of *Escherichia coli*.. Embo J.

[40] Chen DQ, Campbell BC, Purcell AH (1996). A new rickettsia from a herbivorous insect, the pea aphid *Acyrthosiphon pisum* (Harris).. Curr Microbiol.

[41] Gomez-Valero L, Soriano-Navarro M, Perez-Brocal V, Heddi A, Moya A (2004). Coexistence of *Wolbachia* with *Buchnera aphidicola* and a secondary symbiont in the aphid *Cinara cedri*.. J Bacteriol.

[42] Uehara T, Park JT (2007). An anhydro-*N*-acetylmuramyl-L-alanine amidase with broad specificity tethered to the outer membrane of *Escherichia coli*.. J Bacteriol.

[43] Liepinsh E, Genereux C, Dehareng D, Joris B, Otting G (2003). NMR structure of *Citrobacter freundii* AmpD, comparison with bacteriophage T7 lysozyme and homology with PGRP domains.. J Mol Biol.

[44] Genereux C, Dehareng D, Devreese B, Van Beeumen J, Frere JM (2004). Mutational analysis of the catalytic centre of the *Citrobacter freundii* AmpD *N*-acetylmuramyl-L-alanine amidase.. Biochem J.

[45] Darby AC, Cho NH, Fuxelius HH, Westberg J, Andersson SG (2007). Intracellular pathogens go extreme: genome evolution in the Rickettsiales.. Trends Genet.

[46] Horn M, Harzenetter MD, Linner T, Schmid EN, Muller KD (2001). Members of the *Cytophaga*-*Flavobacterium*-*Bacteroides* phylum as intracellular bacteria of acanthamoebae: proposal of '*Candidatus* Amoebophilus asiaticus'.. Environ Microbiol.

[47] Lerat E, Daubin V, Ochman H, Moran NA (2005). Evolutionary origins of genomic repertoires in bacteria.. PLoS Biol.

[48] Jolles P, editor (1996). Lysozymes: Model Enzymes in Biochemistry and Biology..

[49] Gerardo NM, Altincicek B, Anselme C, Atamian H, Barribeau SM (2009). Immunity and other defenses in pea aphids, *Acyrthosiphon pisum*. Genome Biol in press..

[50] Shimodaira H (2002). An approximately unbiased test of phylogenetic tree selection.. Syst Biol.

[51] Shigenobu S, Richards S, Cree AG, Morioka M, Fukatsu T (2009). A full-length cDNA resource for the pea aphid, *Acyrthosiphon pisum*.. Insect Mol Biol in press.

[52] Moran NA (2003). Tracing the evolution of gene loss in obligate bacterial symbionts.. Curr Opin Microbiol.

[53] Serbus LR, Sullivan W (2007). A cellular basis for *Wolbachia* recruitment to the host germline.. PLoS Pathog.

[54] Braendle C, Miura T, Bickel R, Shingleton AW, Kambhampati S (2003). Developmental origin and evolution of bacteriocytes in the aphid-*Buchnera* symbiosis.. PLoS Biol.

[55] Miura T, Braendle C, Shingleton A, Sisk G, Kambhampati S (2003). A comparison of parthenogenetic and sexual embryogenesis of the pea aphid *Acyrthosiphon pisum* (Hemiptera: Aphidoidea).. J Exp Zoolog B Mol Dev Evol.

[56] Houk EJ, Griffiths GW, Hadjokas NE, Beck SD (1977). Peptidoglycan in the cell wall of the primary intracellular symbiote of the pea aphid.. Science.

[57] Gilbert W (1978). Why genes in pieces?. Nature.

[58] Altschul SF, Madden TL, Schaffer AA, Zhang J, Zhang Z (1997). Gapped BLAST and PSI-BLAST: a new generation of protein database search programs.. Nucleic Acids Res.

[59] Gordon D, Desmarais C, Green P (2001). Automated finishing with autofinish.. Genome Res.

[60] Marchler-Bauer A, Bryant SH (2004). CD-Search: protein domain annotations on the fly.. Nucleic Acids Res.

[61] Bendtsen JD, Nielsen H, von Heijne G, Brunak S (2004). Improved prediction of signal peptides: SignalP 3.0.. J Mol Biol.

[62] Katoh K, Kuma K, Toh H, Miyata T (2005). MAFFT version 5: improvement in accuracy of multiple sequence alignment.. Nucleic Acids Res.

[63] Ronquist F, Huelsenbeck JP (2003). MrBayes 3: Bayesian phylogenetic inference under mixed models.. Bioinformatics.

[64] Stamatakis A (2006). RAxML-VI-HPC: maximum likelihood-based phylogenetic analyses with thousands of taxa and mixed models.. Bioinformatics.

[65] Felsenstein J (1989). PHYLIP - Phylogeny Inference Package (Version 3.2).. Cladistics.

[66] Schmidt HA, Strimmer K, Vingron M, von Haeseler A (2002). TREE-PUZZLE: maximum likelihood phylogenetic analysis using quartets and parallel computing.. Bioinformatics.

[67] Abascal F, Zardoya R, Posada D (2005). ProtTest: selection of best-fit models of protein evolution.. Bioinformatics.

