# Supplementary figures and images for: Bacterial Genes in the Aphid Genome: Absence of Functional Gene Transfer from Buchnera to Its Host
Source: PLoS Genet. 2010 Feb 26;6(2):e1000827. doi: 10.1371/journal.pgen.1000827 (PMC2829048; doi:10.1371/journal.pgen.1000827)

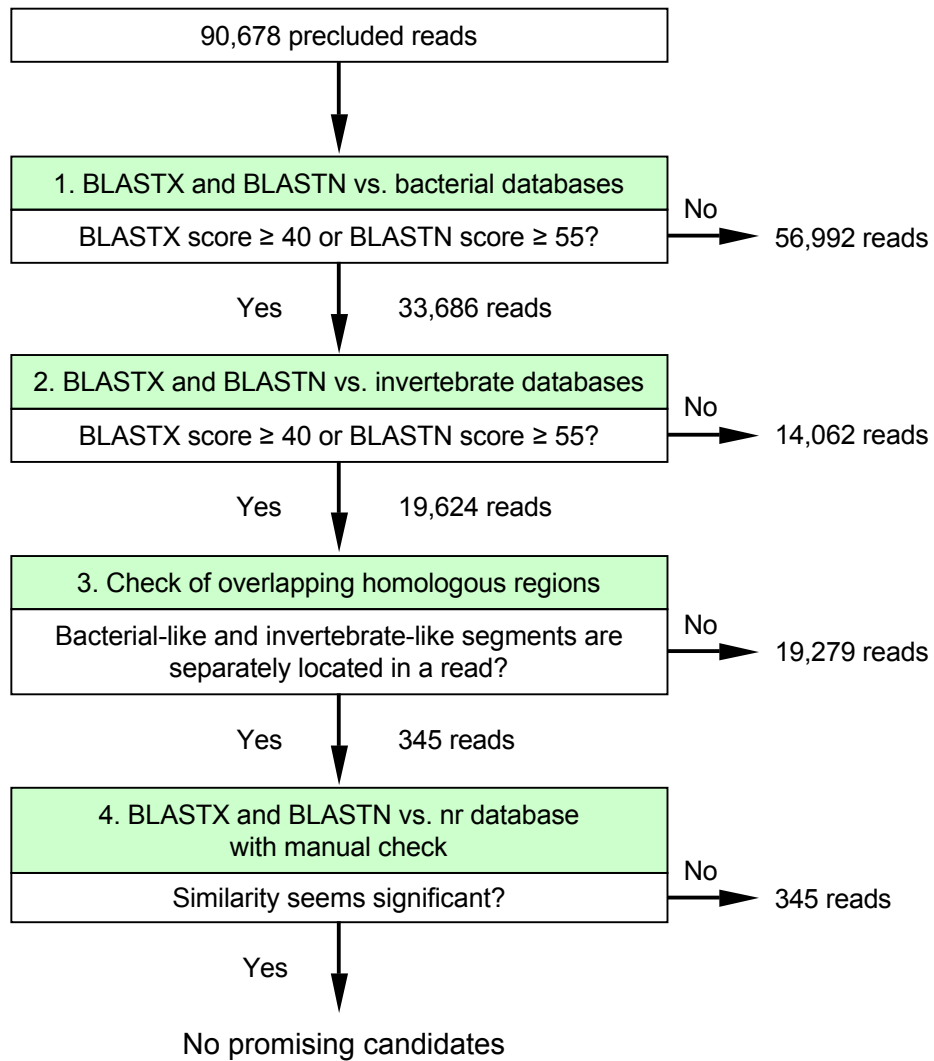

Figure S1

Supplement: Figure S1 — Flow chart of the evaluation of the individual reads precluded from the genome assembly. (0.03 MB PDF) [file pgen.1000827.s001.pdf]

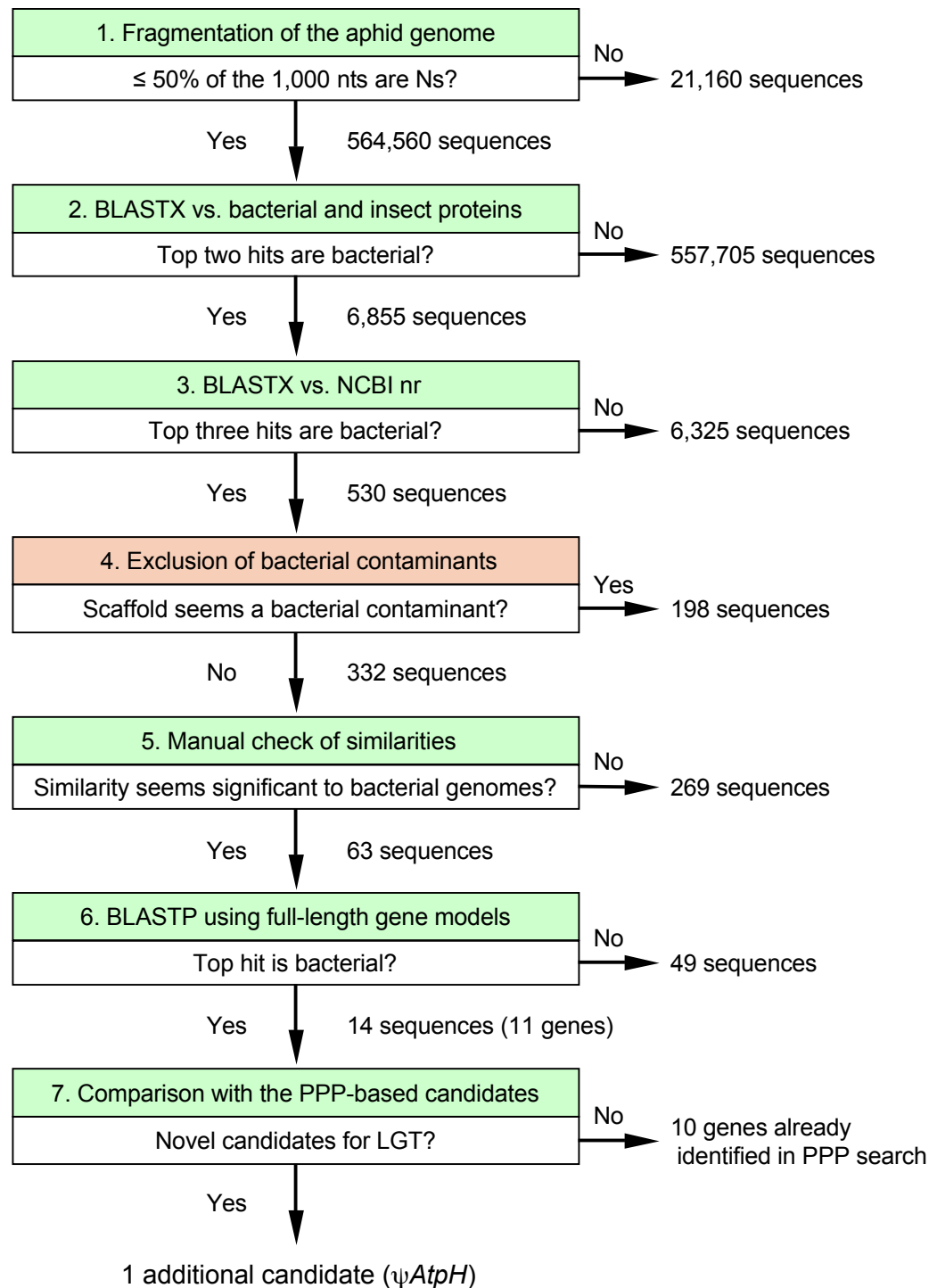

Figure S2

Supplement: Figure S2 — Flow chart of the BLASTX-based screening of the A. pisum genome for LGT candidates. (0.04 MB PDF) [file pgen.1000827.s002.pdf]

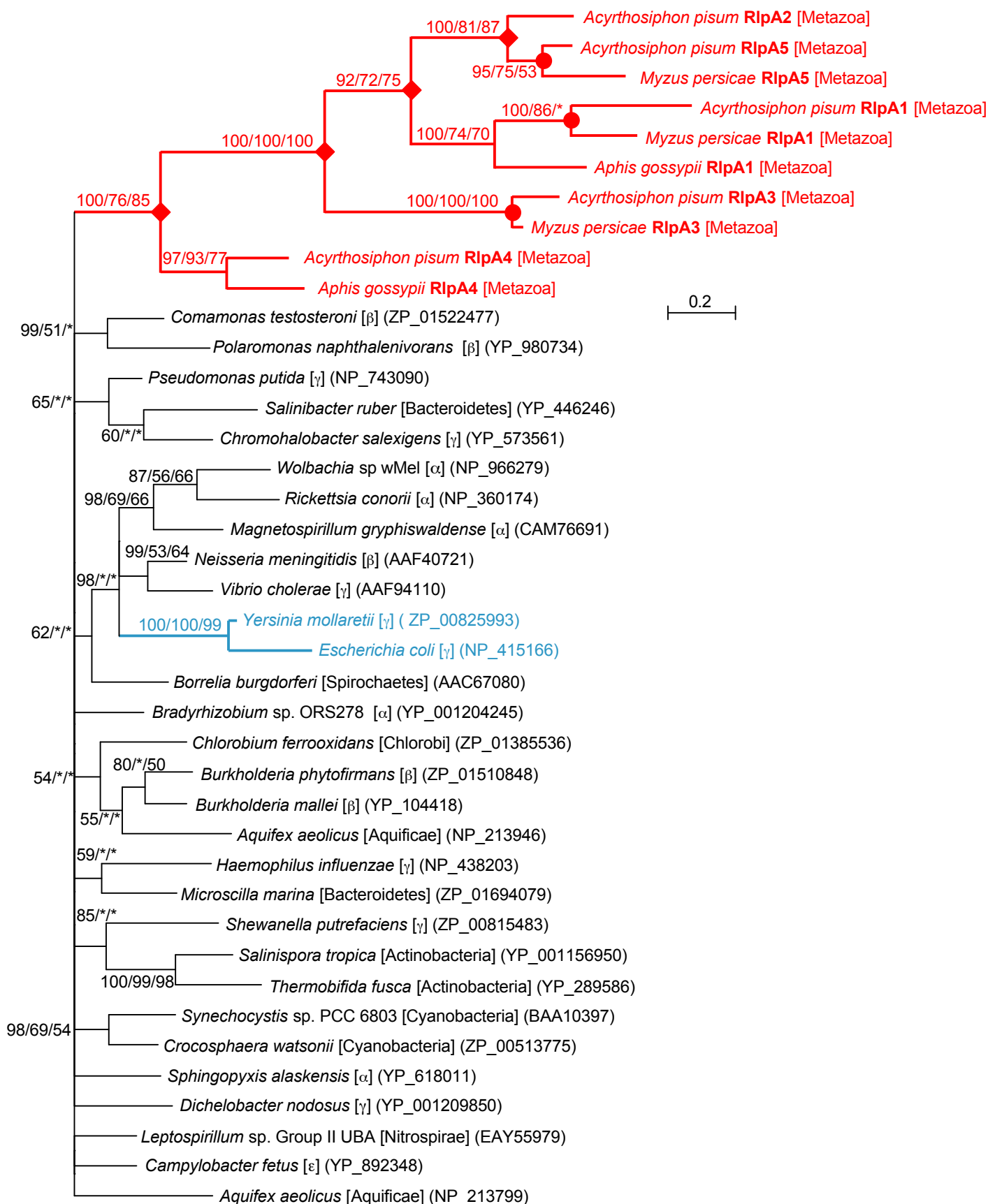

Figure S5

Supplement: Figure S5 — Phylogenetic position of RlpA proteins from three aphid species. The legend is the same as for Figure 12. Circles indicate inferred splits of the ancestors of A. pisum and Myzus persicae. Rhombi indicate inferred duplications of RlpA. Amino acid sequences of RlpA proteins from M. persicae and Aphis gossypii were deduced from assembled EST sequences that were retrieved from NCBI. The accession numbers of the ESTs were DW014944, DW011752, ES221611, ES222724, and DW013043 for M. persicae RlpA1, EE571687 and EE264310 for M. persicae RlpA3, ES221157, EE263538, EE571585, EE262867, and ES220852 for M. persicae RlpA5, DR395894, DR393442, and DR391922 for A. gossypii RlpA1, and DR391796 for A. gossypii RlpA4. (0.06 MB PDF) [file pgen.1000827.s005.pdf]
