# Supplementary material for: Bacterial Genes in the Aphid Genome: Absence of Functional Gene Transfer from Buchnera to Its Host
Source: PLoS Genet. 2010 Feb 26;6(2):e1000827. doi: 10.1371/journal.pgen.1000827 (PMC2829048; doi:10.1371/journal.pgen.1000827)
Supplement: Figure S3 — Alignment of amino acid sequences of DnaEs. Residues conserved in three and two lineages are shaded black and gray, respectively. Triangles and reverse-triangles indicate frameshift deletion and insertion, respectively, in the aphid ψDnaE. Dashes (-) indicate alignment gaps. Asterisks (*) indicate gaps caused by frameshifts. (0.49 MB PDF) [file pgen.1000827.s003.pdf]

```
                                    ▽                  △
A. pisum ψDnaE          : -------------------------------------------------VIKLY**GTAHKLGLKPIIGIA*NFFFNLINNELTKLILLASTQEGYNNLI :  51
Buchnera str. APS DnaE  : MNEPKFIHLHVHSDYSMIDGLSKPEDLVKKAASLNMPAIAITDYNNLYGVIKFY--NMAHKLGLKPIIGITVNFFSDLINNQLTKLTLLASTQEGYKNLI :  98
E. coli DnaE            : MSEPRFVHLRVHSDYSMIDGLAKTAPLVKKAAALGMPALAITDFTNLCGLVKFY--GAGHGAGIKPIVGADFNVQCDLLGDELTHLTVLAANNTGYQNLT :  98

A. pisum ψDnaE          : LLISHTYQKGYVNNYDVTTMKKKWLLKINKINILLSGGCQCEIRKVKLIIYIFIIFY------------------------------------------- : 108
Buchnera str. APS DnaE  : LLISRAYQKGYVHN-NHVTIEKKWFSEINKGLILLSGGPQGELGKVLLNGQSSLISICLSFYQKYFPDSYYLELFRTNRDNEETYLHLAVDLSLSTGVPI : 197
E. coli DnaE            : LLISKAYQRGYGA--AGPIIDRDWLIELNEGLILLSGGRMGDVGRSLLRGNSALVDECVAFYEEHFPDRYFLELIRTGRPDEESYLHAAVELAEARGLPV : 196
```

Figure S3
