# Supplementary material for: Bacterial Genes in the Aphid Genome: Absence of Functional Gene Transfer from Buchnera to Its Host
Source: PLoS Genet. 2010 Feb 26;6(2):e1000827. doi: 10.1371/journal.pgen.1000827 (PMC2829048; doi:10.1371/journal.pgen.1000827)
Supplement: Figure S4 — Alignment of amino acid sequences of AtpHs. Residues conserved in all and two lineages are shaded black and gray, respectively. Dashes (-) indicate alignment gaps. Dots (.) indicate stop codons. (0.43 MB PDF) [file pgen.1000827.s004.pdf]

```
A. pisum ψAtpH          : --------------------------------------------YYIFSGSLSPKYLS.VLIAISGDIINENAINLI.LLAENQGFKILNK--RKFLKLE :  52
Buchnera str. APS AtpH  : MSVADTIARPYAQAIFEIAIENNTIEKWKNILIFIKTIASHKKFKNFLSGSISPKYLSLIFITIGTNIIDENAKNLIKLLSENQRFNILNNIFERFVKLE : 100
E. coli AtpH            : MSEFITVARPYAKAAFDFAVEHQSVERWQDMLAFAAEVTKNEQMAELLSGALAPETLAESFIAVCGEQLDENGQNLIRVMAENGRLNALPDVLEQFIHLR : 100

A. pisum ψAtpH          : ACYKNIIIVGLRSAFSLKEKQINKIRTI.EQSLSRKTKYHCLSPRA------------------------------- :  97
Buchnera str. APS AtpH  : ACYKNIIIVQLKSAFSLKENLINKIRKVLERFFLKKTKIIYKVDPNILNGMIVKVNNTIFDLSAQNHLKQLSDSLNF : 177
E. coli AtpH            : AVSEATAEVDVISAAALSEQQLAKISAAMEKRLSRKVKLNCKIDKSVMAGVIIRAGDMVIDGSVRGRLERLADVLQS : 177
```

Figure S4
