# Supplementary material for: Bacterial Genes in the Aphid Genome: Absence of Functional Gene Transfer from Buchnera to Its Host
Source: PLoS Genet. 2010 Feb 26;6(2):e1000827. doi: 10.1371/journal.pgen.1000827 (PMC2829048; doi:10.1371/journal.pgen.1000827)
Supplement: Table S1 — List of bacteria used to construct bacterial databases. (0.03 MB DOC) [file pgen.1000827.s006.doc]

**Table S1.** **List of bacteria used to construct bacterial databases.**

**Group Accession Species**

Alphaproteobacteria NC_000963 *Rickettsia prowazekii*

Alphaproteobacteria NC_003103 *Rickettsia conorii*

Alphaproteobacteria NC_002696 *Caulobacter crescentus* CB15

Alphaproteobacteria NC_003062 *Agrobacterium tumefaciens* str*.* C58

NC_003063

NC_003064

Alphaproteobacteria NC_003317 *Brucella melitensis*

NC_003318

Alphaproteobacteria NC_002678 *Mesorhizobium loti*

Alphaproteobacteria NC_002978 *Wolbachia pipientis* strain wMel

Alphaproteobacteria NC_006833 *Wolbachia* *pipientis* strain TRS

Gammaproteobacteria NC_002505 *Vibrio cholerae* O1 biovar eltor str. N16961

NC_002506

Gammaproteobacteria NC_002516 *Pseudomonas aeruginosa* PAO1

Gammaproteobacteria NC_003197 *Salmonella typhimurium* LT2

Gammaproteobacteria NC_003919 *Xanthomonas axonopodis* pv. citri str. 306

Gammaproteobacteria NC_004347 *Shewanella oneidensis* MR-1

Gammaproteobacteria NC_007712 *Sodalis glossinidius* str. 'morsitans'

Gammaproteobacteria NC_004344 *Wigglesworthia glossinidia*

Gammaproteobacteria NC_000913 *Escherichia coli* K12

Gammaproteobacteria NC_000907 *Haemophilus influenzae* Rd KW20

Gammaproteobacteria NC_004061 *Buchnera aphidicola* str. Sg

Gammaproteobacteria NC_008513 *Buchnera aphidicola* str. Cc

Gammaproteobacteria NC_004545 *Buchnera aphidicola* str. Bp

Gammaproteobacteria NC_002528 *Buchnera aphidicola* str. APS

Gammaproteobacteria NC_007292 *Blochmannia pennsylvanicus* str. BPEN

Gammaproteobacteria NC_005061 *Blochmannia floridanus*

Gammaproteobacteria NC_007984 *Baumannia cicadellinicola* str. Hc

Gammaproteobacteria NC_004547 *Erwinia carotovora* subsp. atroseptica SCRI1043

Gammaproteobacteria NC_005126 *Photorhabdus luminescens* subsp. laumondii TTO1

Gammaproteobacteria NC_002695 *Escherichia coli* O157:H7 str. Sakai

Gammaproteobacteria NC_003143 *Yersinia pestis* CO92

Gammaproteobacteria NC_008512 *Carsonella ruddii* PV

Betaproteobacteria NC_002946 *Neisseria gonorrhoeae* FA 1090

Betaproteobacteria NC_003295 *Ralstonia solanacearum*

Betaproteobacteria NC_004757 *Nitrosomonas europaea* ATCC 1971

Firmicutes NC_002951 *Staphylococcus aureus* subsp. aureus Mu50

Firmicutes NC_006582 *Bacillus clausii* KSM-K16

Firmicutes NC_000964 *Bacillus subtilis* subsp. subtilis str. 168
