# Supplementary material for: Bacterial Genes in the Aphid Genome: Absence of Functional Gene Transfer from Buchnera to Its Host
Source: PLoS Genet. 2010 Feb 26;6(2):e1000827. doi: 10.1371/journal.pgen.1000827 (PMC2829048; doi:10.1371/journal.pgen.1000827)
Supplement: Table S2 — List of bacteria and archaea used to construct a protein database (for the BLASTX-based screening). (0.07 MB DOC) [file pgen.1000827.s007.doc]

**Table S2.** **List of bacteria and archaea used to construct a protein database (for the BLASTX-based screening).**

Acaryochloris marina MBIC11017

Acholeplasma laidlawii PG-8A

Acidiphilium cryptum JF-5

Acidobacteria bacterium Ellin345

Acidothermus cellulolyticus 11B

Acidovorax avenae subsp. citrulli AAC00-1

Acidovorax sp. JS42

Acinetobacter baumannii

Acinetobacter baumannii ATCC 17978

Acinetobacter baumannii AYE

Acinetobacter sp. ADP1

Actinobacillus pleuropneumoniae L20

Actinobacillus pleuropneumoniae serovar 3 str. JL03

Actinobacillus succinogenes 130Z

Aeromonas hydrophila subsp. hydrophila ATCC 7966

Aeromonas salmonicida subsp. salmonicida A449

Aeropyrum pernix K1

Agrobacterium tumefaciens str. C58

Alcanivorax borkumensis SK2

Alkalilimnicola ehrlichei MLHE-1

Alkaliphilus metalliredigens QYMF

Alkaliphilus oremlandii OhILAs

Anabaena variabilis ATCC 29413

Anaeromyxobacter dehalogenans 2CP-C

Anaeromyxobacter sp. Fw109-5

Anaplasma marginale str. St. Maries

Anaplasma phagocytophilum HZ

Aquifex aeolicus VF5

Archaeoglobus fulgidus DSM 4304

Arcobacter butzleri RM4018

Arthrobacter aurescens TC1

Arthrobacter sp. FB24

Aster yellows witches'-broom phytoplasma AYWB

Azoarcus sp. BH72

Azoarcus sp. EbN1

Azorhizobium caulinodans ORS 571

Bacillus amyloliquefaciens FZB42

Bacillus anthracis str. 'Ames Ancestor'

Bacillus anthracis str. Ames

Bacillus anthracis str. Sterne

Bacillus cereus ATCC 10987

Bacillus cereus ATCC 14579

Bacillus cereus E33L

Bacillus cereus subsp. cytotoxis NVH 391-98

Bacillus clausii KSM-K16

Bacillus halodurans C-125

Bacillus licheniformis ATCC 14580

Bacillus pumilus SAFR-032

Bacillus subtilis subsp. subtilis str. 168

Bacillus thuringiensis serovar konkukian str. 97-27

Bacillus thuringiensis str. Al Hakam

Bacillus weihenstephanensis KBAB4

Bacteroides fragilis NCTC 9343

Bacteroides fragilis YCH46

Bacteroides thetaiotaomicron VPI-5482

Bacteroides vulgatus ATCC 8482

Bartonella bacilliformis KC583

Bartonella henselae str. Houston-1

Bartonella quintana str. Toulouse

Bartonella tribocorum CIP 105476

Baumannia cicadellinicola str. Hc (Homalodisca coagulata)

Bdellovibrio bacteriovorus HD100

Beijerinckia indica subsp. indica ATCC 9039

Bifidobacterium adolescentis ATCC 15703

Bifidobacterium longum NCC2705

Bordetella avium 197N

Bordetella bronchiseptica RB50

Bordetella parapertussis 12822

Bordetella pertussis Tohama I

Bordetella petrii DSM 12804

Borrelia afzelii PKo

Borrelia burgdorferi B31

Borrelia garinii PBi

Bradyrhizobium japonicum USDA 110

Bradyrhizobium sp. BTAi1

Bradyrhizobium sp. ORS278

Brucella abortus biovar 1 str. 9-941

Brucella canis ATCC 23365

Brucella melitensis 16M

Brucella melitensis biovar Abortus 2308

Brucella ovis ATCC 25840

Brucella suis 1330

Brucella suis ATCC 23445

Buchnera aphidicola str. APS (Acyrthosiphon pisum)

Buchnera aphidicola str. Bp (Baizongia pistaciae)

Buchnera aphidicola str. Cc (Cinara cedri)

Buchnera aphidicola str. Sg (Schizaphis graminum)

Burkholderia ambifaria AMMD

Burkholderia ambifaria MC40-6

Burkholderia cenocepacia AU 1054

Burkholderia cenocepacia HI2424

Burkholderia cenocepacia MC0-3

Burkholderia mallei ATCC 23344

Burkholderia mallei NCTC 10229

Burkholderia mallei NCTC 10247

Burkholderia mallei SAVP1

Burkholderia multivorans ATCC 17616

Burkholderia pseudomallei 1106a

Burkholderia pseudomallei 1710b

Burkholderia pseudomallei 668

Burkholderia pseudomallei K96243

Burkholderia sp. 383

Burkholderia thailandensis E264

Burkholderia vietnamiensis G4

Burkholderia xenovorans LB400

Caldicellulosiruptor saccharolyticus DSM 8903

Caldivirga maquilingensis IC-167

Campylobacter concisus 13826

Campylobacter curvus 525.92

Campylobacter fetus subsp. fetus 82-40

Campylobacter hominis ATCC BAA-381

Campylobacter jejuni RM1221

Campylobacter jejuni subsp. doylei 269.97

Campylobacter jejuni subsp. jejuni 81-176

Campylobacter jejuni subsp. jejuni 81116

Campylobacter jejuni subsp. jejuni NCTC 11168

Candidatus Blochmannia floridanus

Candidatus Blochmannia pennsylvanicus str. BPEN

Candidatus Carsonella ruddii PV

Candidatus Desulforudis audaxviator MP104C

Candidatus Korarchaeum cryptofilum OPF8

Candidatus Methanoregula boonei 6A8

Candidatus Pelagibacter ubique HTCC1062

Candidatus Protochlamydia amoebophila UWE25

Candidatus Ruthia magnifica str. Cm (Calyptogena magnifica)

Candidatus Sulcia muelleri GWSS

Candidatus Vesicomyosocius okutanii HA

Carboxydothermus hydrogenoformans Z-2901

Caulobacter crescentus CB15

Caulobacter sp. K31

Chlamydia muridarum Nigg

Chlamydia trachomatis 434/Bu

Chlamydia trachomatis A/HAR-13

Chlamydia trachomatis D/UW-3/CX

Chlamydia trachomatis L2b/UCH-1/proctitis

Chlamydophila abortus S26/3

Chlamydophila caviae GPIC

Chlamydophila felis Fe/C-56

Chlamydophila pneumoniae AR39

Chlamydophila pneumoniae CWL029

Chlamydophila pneumoniae J138

Chlamydophila pneumoniae TW-183

Chlorobium chlorochromatii CaD3

Chlorobium phaeobacteroides DSM 266

Chlorobium tepidum TLS

Chloroflexus aurantiacus J-10-fl

Chromobacterium violaceum ATCC 12472

Chromohalobacter salexigens DSM 3043

Citrobacter koseri ATCC BAA-895

Clavibacter michiganensis subsp. michiganensis NCPPB 382

Clavibacter michiganensis subsp. sepedonicus

Clostridium acetobutylicum ATCC 824

Clostridium beijerinckii NCIMB 8052

Clostridium botulinum A str. ATCC 19397

Clostridium botulinum A str. ATCC 3502

Clostridium botulinum A str. Hall

Clostridium botulinum A3 str. Loch Maree

Clostridium botulinum B str. Eklund 17B

Clostridium botulinum B1 str. Okra

Clostridium botulinum F str. Langeland

Clostridium difficile 630

Clostridium kluyveri DSM 555

Clostridium novyi NT

Clostridium perfringens ATCC 13124

Clostridium perfringens SM101

Clostridium perfringens phage phiSM101

Clostridium perfringens str. 13

Clostridium phytofermentans ISDg

Clostridium tetani E88

Clostridium thermocellum ATCC 27405

Colwellia psychrerythraea 34H

Corynebacterium diphtheriae NCTC 13129

Corynebacterium efficiens YS-314

Corynebacterium glutamicum ATCC 13032

Corynebacterium glutamicum R

Corynebacterium jeikeium K411

Corynebacterium urealyticum DSM 7109

Coxiella burnetii Dugway 5J108-111

Coxiella burnetii RSA 331

Coxiella burnetii RSA 493

Cyanothece sp. ATCC 51142

Cytophaga hutchinsonii ATCC 33406

Dechloromonas aromatica RCB

Dehalococcoides ethenogenes 195

Dehalococcoides sp. BAV1

Dehalococcoides sp. CBDB1

Deinococcus geothermalis DSM 11300

Deinococcus radiodurans R1

Delftia acidovorans SPH-1

Desulfitobacterium hafniense Y51

Desulfococcus oleovorans Hxd3

Desulfotalea psychrophila LSv54

Desulfotomaculum reducens MI-1

Desulfovibrio desulfuricans G20

Desulfovibrio vulgaris subsp. vulgaris DP4

Desulfovibrio vulgaris subsp. vulgaris str. Hildenborough

Dichelobacter nodosus VCS1703A

Dinoroseobacter shibae DFL 12

Ehrlichia canis str. Jake

Ehrlichia chaffeensis str. Arkansas

Ehrlichia ruminantium str. Gardel

Ehrlichia ruminantium str. Welgevonden

Enterobacter sakazakii ATCC BAA-894

Enterobacter sp. 638

Enterobacteria phage Fels-2

Enterococcus faecalis V583

Erwinia carotovora subsp. atroseptica SCRI1043

Erythrobacter litoralis HTCC2594

Escherichia coli 536

Escherichia coli APEC O1

Escherichia coli ATCC 8739

Escherichia coli CFT073

Escherichia coli E24377A

Escherichia coli HS

Escherichia coli O157:H7 EDL933

Escherichia coli O157:H7 str. Sakai

Escherichia coli SECEC SMS-3-5

Escherichia coli UTI89

Escherichia coli W3110

Escherichia coli str. K-12 substr. DH10B

Escherichia coli str. K-12 substr. MG1655

Exiguobacterium sibiricum 255-15

Fervidobacterium nodosum Rt17-B1

Finegoldia magna ATCC 29328

Flavobacterium johnsoniae UW101

Flavobacterium psychrophilum JIP02/86

Francisella philomiragia subsp. philomiragia ATCC 25017

Francisella tularensis subsp. holarctica

Francisella tularensis subsp. holarctica FTNF002-00

Francisella tularensis subsp. holarctica OSU18

Francisella tularensis subsp. mediasiatica FSC147

Francisella tularensis subsp. novicida U112

Francisella tularensis subsp. tularensis FSC198

Francisella tularensis subsp. tularensis SCHU S4

Francisella tularensis subsp. tularensis WY96-3418

Frankia alni ACN14a

Frankia sp. CcI3

Frankia sp. EAN1pec

Fusobacterium nucleatum subsp. nucleatum ATCC 25586

Geobacillus kaustophilus HTA426

Geobacillus thermodenitrificans NG80-2

Geobacter metallireducens GS-15

Geobacter sulfurreducens PCA

Geobacter uraniireducens Rf4

Gloeobacter violaceus PCC 7421

Gluconacetobacter diazotrophicus PAl 5

Gluconobacter oxydans 621H

Gramella forsetii KT0803

Granulibacter bethesdensis CGDNIH1

Haemophilus ducreyi 35000HP

Haemophilus influenzae 86-028NP

Haemophilus influenzae PittEE

Haemophilus influenzae PittGG

Haemophilus influenzae Rd KW20

Haemophilus somnus 129PT

Haemophilus somnus 2336

Hahella chejuensis KCTC 2396

Haloarcula marismortui ATCC 43049

Halobacterium salinarum R1

Halobacterium sp. NRC-1

Haloquadratum walsbyi DSM 16790

Halorhodospira halophila SL1

Helicobacter acinonychis str. Sheeba

Helicobacter hepaticus ATCC 51449

Helicobacter pylori 26695

Helicobacter pylori HPAG1

Helicobacter pylori J99

Heliobacterium modesticaldum Ice1

Herminiimonas arsenicoxydans

Herpetosiphon aurantiacus ATCC 23779

Hyperthermus butylicus DSM 5456

Hyphomonas neptunium ATCC 15444

Idiomarina loihiensis L2TR

Ignicoccus hospitalis KIN4/I

Jannaschia sp. CCS1

Janthinobacterium sp. Marseille

Kineococcus radiotolerans SRS30216

Klebsiella pneumoniae subsp. pneumoniae MGH 78578

Lactobacillus acidophilus NCFM

Lactobacillus brevis ATCC 367

Lactobacillus casei ATCC 334

Lactobacillus delbrueckii subsp. bulgaricus ATCC 11842

Lactobacillus delbrueckii subsp. bulgaricus ATCC BAA-365

Lactobacillus fermentum IFO 3956

Lactobacillus gasseri ATCC 33323

Lactobacillus helveticus DPC 4571

Lactobacillus johnsonii NCC 533

Lactobacillus phage Sal1

Lactobacillus phage Sal2

Lactobacillus phage Sal3

Lactobacillus phage Sal4

Lactobacillus plantarum WCFS1

Lactobacillus reuteri F275

Lactobacillus sakei subsp. sakei 23K

Lactobacillus salivarius UCC118

Lactococcus lactis subsp. cremoris MG1363

Lactococcus lactis subsp. cremoris SK11

Lactococcus lactis subsp. lactis Il1403

Lawsonia intracellularis PHE/MN1-00

Legionella pneumophila str. Corby

Legionella pneumophila str. Lens

Legionella pneumophila str. Paris

Legionella pneumophila subsp. pneumophila str. Philadelphia 1

Leifsonia xyli subsp. xyli str. CTCB07

Leptospira biflexa serovar Patoc strain 'Patoc 1 (Paris)'

Leptospira borgpetersenii serovar Hardjo-bovis JB197

Leptospira borgpetersenii serovar Hardjo-bovis L550

Leptospira interrogans serovar Copenhageni str. Fiocruz L1-130

Leptospira interrogans serovar Lai str. 56601

Leptothrix cholodnii SP-6

Leuconostoc citreum KM20

Leuconostoc mesenteroides subsp. mesenteroides ATCC 8293

Listeria innocua Clip11262

Listeria monocytogenes EGD-e

Listeria monocytogenes str. 4b F2365

Listeria welshimeri serovar 6b str. SLCC5334

Lysinibacillus sphaericus C3-41

Magnetococcus sp. MC-1

Magnetospirillum magneticum AMB-1

Mannheimia succiniciproducens MBEL55E

Maricaulis maris MCS10

Marinobacter aquaeolei VT8

Marinomonas sp. MWYL1

Mesoplasma florum L1

Mesorhizobium loti MAFF303099

Mesorhizobium sp. BNC1

Metallosphaera sedula DSM 5348

Methanobrevibacter smithii ATCC 35061

Methanocaldococcus jannaschii DSM 2661

Methanococcoides burtonii DSM 6242

Methanococcus aeolicus Nankai-3

Methanococcus maripaludis C5

Methanococcus maripaludis C6

Methanococcus maripaludis C7

Methanococcus maripaludis S2

Methanococcus vannielii SB

Methanocorpusculum labreanum Z

Methanoculleus marisnigri JR1

Methanopyrus kandleri AV19

Methanosaeta thermophila PT

Methanosarcina acetivorans C2A

Methanosarcina barkeri str. Fusaro

Methanosarcina mazei Go1

Methanosphaera stadtmanae DSM 3091

Methanospirillum hungatei JF-1

Methanothermobacter thermautotrophicus str. Delta H

Methylibium petroleiphilum PM1

Methylobacillus flagellatus KT

Methylobacterium extorquens PA1

Methylobacterium radiotolerans JCM 2831

Methylobacterium sp. 4-46

Methylococcus capsulatus str. Bath

Microcystis aeruginosa NIES-843

Moorella thermoacetica ATCC 39073

Mycobacterium abscessus

Mycobacterium avium 104

Mycobacterium avium subsp. paratuberculosis K-10

Mycobacterium bovis AF2122/97

Mycobacterium bovis BCG str. Pasteur 1173P2

Mycobacterium gilvum PYR-GCK

Mycobacterium leprae TN

Mycobacterium marinum M

Mycobacterium smegmatis str. MC2 155

Mycobacterium sp. JLS

Mycobacterium sp. KMS

Mycobacterium sp. MCS

Mycobacterium tuberculosis CDC1551

Mycobacterium tuberculosis F11

Mycobacterium tuberculosis H37Ra

Mycobacterium tuberculosis H37Rv

Mycobacterium ulcerans Agy99

Mycobacterium vanbaalenii PYR-1

Mycoplasma agalactiae PG2

Mycoplasma capricolum subsp. capricolum ATCC 27343

Mycoplasma gallisepticum R

Mycoplasma genitalium G37

Mycoplasma hyopneumoniae 232

Mycoplasma hyopneumoniae 7448

Mycoplasma hyopneumoniae J

Mycoplasma mobile 163K

Mycoplasma mycoides subsp. mycoides SC str. PG1

Mycoplasma penetrans HF-2

Mycoplasma pneumoniae M129

Mycoplasma pulmonis UAB CTIP

Mycoplasma synoviae 53

Myxococcus xanthus DK 1622

Nanoarchaeum equitans Kin4-M

Natronomonas pharaonis DSM 2160

Neisseria gonorrhoeae FA 1090

Neisseria meningitidis 053442

Neisseria meningitidis FAM18

Neisseria meningitidis MC58

Neisseria meningitidis Z2491

Neorickettsia sennetsu str. Miyayama

Nitratiruptor sp. SB155-2

Nitrobacter hamburgensis X14

Nitrobacter winogradskyi Nb-255

Nitrosococcus oceani ATCC 19707

Nitrosomonas europaea ATCC 19718

Nitrosomonas eutropha C91

Nitrosopumilus maritimus SCM1

Nitrosospira multiformis ATCC 25196

Nocardia farcinica IFM 10152

Nocardioides sp. JS614

Nostoc sp. PCC 7120

Novosphingobium aromaticivorans DSM 12444

Oceanobacillus iheyensis HTE831

Ochrobactrum anthropi ATCC 49188

Oenococcus oeni PSU-1

Onion yellows phytoplasma OY-M

Opitutus terrae PB90-1

Orientia tsutsugamushi Boryong

Parabacteroides distasonis ATCC 8503

Paracoccus denitrificans PD1222

Parvibaculum lavamentivorans DS-1

Pasteurella multocida subsp. multocida str. Pm70

Pediococcus pentosaceus ATCC 25745

Pelobacter carbinolicus DSM 2380

Pelobacter propionicus DSM 2379

Pelodictyon luteolum DSM 273

Pelotomaculum thermopropionicum SI

Petrotoga mobilis SJ95

Phage Gifsy-1

Phage Gifsy-2

Photobacterium profundum SS9

Photorhabdus luminescens subsp. laumondii TTO1

Picrophilus torridus DSM 9790

Polaromonas naphthalenivorans CJ2

Polaromonas sp. JS666

Polynucleobacter necessarius STIR1

Polynucleobacter sp. QLW-P1DMWA-1

Porphyromonas gingivalis W83

Prochlorococcus marinus str. AS9601

Prochlorococcus marinus str. MIT 9211

Prochlorococcus marinus str. MIT 9215

Prochlorococcus marinus str. MIT 9301

Prochlorococcus marinus str. MIT 9303

Prochlorococcus marinus str. MIT 9312

Prochlorococcus marinus str. MIT 9313

Prochlorococcus marinus str. MIT 9515

Prochlorococcus marinus str. NATL1A

Prochlorococcus marinus str. NATL2A

Prochlorococcus marinus subsp. marinus str. CCMP1375

Prochlorococcus marinus subsp. pastoris str. CCMP1986

Propionibacterium acnes KPA171202

Prosthecochloris vibrioformis DSM 265

Pseudoalteromonas atlantica T6c

Pseudoalteromonas haloplanktis TAC125

Pseudomonas aeruginosa PA7

Pseudomonas aeruginosa PAO1

Pseudomonas aeruginosa UCBPP-PA14

Pseudomonas entomophila L48

Pseudomonas fluorescens Pf-5

Pseudomonas fluorescens PfO-1

Pseudomonas mendocina ymp

Pseudomonas putida F1

Pseudomonas putida GB-1

Pseudomonas putida KT2440

Pseudomonas putida W619

Pseudomonas stutzeri A1501

Pseudomonas syringae pv. phaseolicola 1448A

Pseudomonas syringae pv. syringae B728a

Pseudomonas syringae pv. tomato str. DC3000

Psychrobacter arcticus 273-4

Psychrobacter cryohalolentis K5

Psychrobacter sp. PRwf-1

Psychromonas ingrahamii 37

Pyrobaculum aerophilum str. IM2

Pyrobaculum arsenaticum DSM 13514

Pyrobaculum calidifontis JCM 11548

Pyrobaculum islandicum DSM 4184

Pyrococcus abyssi GE5

Pyrococcus furiosus DSM 3638

Pyrococcus horikoshii OT3

Ralstonia eutropha H16

Ralstonia eutropha JMP134

Ralstonia metallidurans CH34

Ralstonia solanacearum GMI1000

Renibacterium salmoninarum ATCC 33209

Rhizobium etli CFN 42

Rhizobium leguminosarum bv. viciae 3841

Rhodobacter sphaeroides 2.4.1

Rhodobacter sphaeroides ATCC 17025

Rhodobacter sphaeroides ATCC 17029

Rhodococcus sp. RHA1

Rhodoferax ferrireducens T118

Rhodopirellula baltica SH 1

Rhodopseudomonas palustris BisA53

Rhodopseudomonas palustris BisB18

Rhodopseudomonas palustris BisB5

Rhodopseudomonas palustris CGA009

Rhodopseudomonas palustris HaA2

Rhodospirillum rubrum ATCC 11170

Rickettsia akari str. Hartford

Rickettsia bellii OSU 85-389

Rickettsia bellii RML369-C

Rickettsia canadensis str. McKiel

Rickettsia conorii str. Malish 7

Rickettsia felis URRWXCal2

Rickettsia massiliae MTU5

Rickettsia prowazekii str. Madrid E

Rickettsia rickettsii str. 'Sheila Smith'

Rickettsia rickettsii str. Iowa

Rickettsia typhi str. Wilmington

Roseiflexus castenholzii DSM 13941

Roseiflexus sp. RS-1

Roseobacter denitrificans OCh 114

Rubrobacter xylanophilus DSM 9941

Saccharophagus degradans 2-40

Saccharopolyspora erythraea NRRL 2338

Saccharopolyspora erythraea prophage pSE101

Saccharopolyspora erythraea prophage pSE211

Salinibacter ruber DSM 13855

Salinispora arenicola CNS-205

Salinispora tropica CNB-440

Salmonella enterica subsp. arizonae serovar 62:z4,z23:--

Salmonella enterica subsp. enterica serovar Choleraesuis str. SC-B67

Salmonella enterica subsp. enterica serovar Paratyphi A str. ATCC 9150

Salmonella enterica subsp. enterica serovar Paratyphi B str. SPB7

Salmonella enterica subsp. enterica serovar Typhi Ty2

Salmonella enterica subsp. enterica serovar Typhi str. CT18

Salmonella phage Fels-1

Salmonella typhimurium LT2

Serratia proteamaculans 568

Shewanella amazonensis SB2B

Shewanella baltica OS155

Shewanella baltica OS185

Shewanella baltica OS195

Shewanella denitrificans OS217

Shewanella frigidimarina NCIMB 400

Shewanella halifaxensis HAW-EB4

Shewanella loihica PV-4

Shewanella oneidensis MR-1

Shewanella pealeana ATCC 700345

Shewanella putrefaciens CN-32

Shewanella sediminis HAW-EB3

Shewanella sp. ANA-3

Shewanella sp. MR-4

Shewanella sp. MR-7

Shewanella sp. W3-18-1

Shewanella woodyi ATCC 51908

Shigella boydii CDC 3083-94

Shigella boydii Sb227

Shigella dysenteriae Sd197

Shigella flexneri 2a str. 2457T

Shigella flexneri 2a str. 301

Shigella flexneri 5 str. 8401

Shigella sonnei Ss046

Silicibacter pomeroyi DSS-3

Silicibacter sp. TM1040

Sinorhizobium medicae WSM419

Sinorhizobium meliloti 1021

Sodalis glossinidius str. 'morsitans'

Solibacter usitatus Ellin6076

Sorangium cellulosum 'So ce 56'

Sphingomonas wittichii RW1

Sphingopyxis alaskensis RB2256

Staphylococcus aureus RF122

Staphylococcus aureus subsp. aureus COL

Staphylococcus aureus subsp. aureus JH1

Staphylococcus aureus subsp. aureus JH9

Staphylococcus aureus subsp. aureus MRSA252

Staphylococcus aureus subsp. aureus MSSA476

Staphylococcus aureus subsp. aureus MW2

Staphylococcus aureus subsp. aureus Mu3

Staphylococcus aureus subsp. aureus Mu50

Staphylococcus aureus subsp. aureus N315

Staphylococcus aureus subsp. aureus NCTC 8325

Staphylococcus aureus subsp. aureus USA300

Staphylococcus aureus subsp. aureus USA300_TCH1516

Staphylococcus aureus subsp. aureus str. Newman

Staphylococcus epidermidis ATCC 12228

Staphylococcus epidermidis RP62A

Staphylococcus haemolyticus JCSC1435

Staphylococcus saprophyticus subsp. saprophyticus ATCC 15305

Staphylothermus marinus F1

Streptococcus agalactiae 2603V/R

Streptococcus agalactiae A909

Streptococcus agalactiae NEM316

Streptococcus gordonii str. Challis substr. CH1

Streptococcus mutans UA159

Streptococcus phage 10270.1

Streptococcus phage 10270.2

Streptococcus phage 10270.3

Streptococcus phage 10270.4

Streptococcus phage 10270.5

Streptococcus phage 10750.1

Streptococcus phage 10750.2

Streptococcus phage 10750.3

Streptococcus phage 10750.4

Streptococcus phage 2096.1

Streptococcus phage 2096.2

Streptococcus phage 370.1

Streptococcus phage 370.2

Streptococcus phage 370.3

Streptococcus phage 370.4

Streptococcus phage 6180.1

Streptococcus phage 9428.3

Streptococcus phage 9429.1

Streptococcus phage 9429.2

Streptococcus pneumoniae CGSP14

Streptococcus pneumoniae D39

Streptococcus pneumoniae Hungary19A-6

Streptococcus pneumoniae R6

Streptococcus pneumoniae TIGR4

Streptococcus pyogenes M1 GAS

Streptococcus pyogenes MGAS10270

Streptococcus pyogenes MGAS10394

Streptococcus pyogenes MGAS10750

Streptococcus pyogenes MGAS2096

Streptococcus pyogenes MGAS315

Streptococcus pyogenes MGAS5005

Streptococcus pyogenes MGAS6180

Streptococcus pyogenes MGAS8232

Streptococcus pyogenes MGAS9429

Streptococcus pyogenes SSI-1

Streptococcus pyogenes phage 5005.1

Streptococcus pyogenes phage 5005.2

Streptococcus pyogenes phage 5005.3

Streptococcus pyogenes phage 6180.2

Streptococcus pyogenes phage 6180.3

Streptococcus pyogenes phage 6180.4

Streptococcus pyogenes str. Manfredo

Streptococcus sanguinis SK36

Streptococcus suis 05ZYH33

Streptococcus suis 98HAH33

Streptococcus thermophilus CNRZ1066

Streptococcus thermophilus LMD-9

Streptococcus thermophilus LMG 18311

Streptomyces avermitilis MA-4680

Streptomyces coelicolor A3(2)

Streptomyces griseus subsp. griseus NBRC 13350

Sulfolobus acidocaldarius DSM 639

Sulfolobus solfataricus P2

Sulfolobus tokodaii str. 7

Sulfurimonas denitrificans DSM 1251

Sulfurovum sp. NBC37-1

Symbiobacterium thermophilum IAM 14863

Synechococcus elongatus PCC 6301

Synechococcus elongatus PCC 7942

Synechococcus sp. CC9311

Synechococcus sp. CC9605

Synechococcus sp. CC9902

Synechococcus sp. JA-2-3B'a(2-13)

Synechococcus sp. JA-3-3Ab

Synechococcus sp. PCC 7002

Synechococcus sp. RCC307

Synechococcus sp. WH 7803

Synechococcus sp. WH 8102

Synechocystis sp. PCC 6803

Syntrophobacter fumaroxidans MPOB

Syntrophomonas wolfei subsp. wolfei str. Goettingen

Syntrophus aciditrophicus SB

Thermoanaerobacter pseudethanolicus ATCC 33223

Thermoanaerobacter sp. X514

Thermoanaerobacter tengcongensis MB4

Thermobifida fusca YX

Thermococcus kodakarensis KOD1

Thermofilum pendens Hrk 5

Thermoplasma acidophilum DSM 1728

Thermoplasma volcanium GSS1

Thermoproteus neutrophilus V24Sta

Thermosipho melanesiensis BI429

Thermosynechococcus elongatus BP-1

Thermotoga lettingae TMO

Thermotoga maritima MSB8

Thermotoga petrophila RKU-1

Thermus thermophilus HB27

Thermus thermophilus HB8

Thiobacillus denitrificans ATCC 25259

Thiomicrospira crunogena XCL-2

Treponema denticola ATCC 35405

Treponema pallidum subsp. pallidum str. Nichols

Trichodesmium erythraeum IMS101

Tropheryma whipplei TW08/27

Tropheryma whipplei str. Twist

Ureaplasma parvum serovar 3 str. ATCC 27815

Ureaplasma parvum serovar 3 str. ATCC 700970

Verminephrobacter eiseniae EF01-2

Vibrio cholerae O1 biovar eltor str. N16961

Vibrio cholerae O395

Vibrio fischeri ES114

Vibrio harveyi ATCC BAA-1116

Vibrio parahaemolyticus RIMD 2210633

Vibrio vulnificus CMCP6

Vibrio vulnificus YJ016

Wigglesworthia glossinidia endosymbiont of Glossina brevipalpis

Wolbachia endosymbiont of Drosophila melanogaster

Wolbachia endosymbiont strain TRS of Brugia malayi

Wolinella succinogenes DSM 1740

Xanthobacter autotrophicus Py2

Xanthomonas axonopodis pv. citri str. 306

Xanthomonas campestris pv. campestris str. 8004

Xanthomonas campestris pv. campestris str. ATCC 33913

Xanthomonas campestris pv. vesicatoria str. 85-10

Xanthomonas oryzae pv. oryzae KACC10331

Xanthomonas oryzae pv. oryzae MAFF 311018

Xylella fastidiosa 9a5c

Xylella fastidiosa M12

Xylella fastidiosa M23

Xylella fastidiosa Temecula1

Yersinia enterocolitica subsp. enterocolitica 8081

Yersinia pestis Angola

Yersinia pestis Antiqua

Yersinia pestis CO92

Yersinia pestis KIM

Yersinia pestis Nepal516

Yersinia pestis Pestoides F

Yersinia pestis biovar Microtus str. 91001

Yersinia pseudotuberculosis IP 31758

Yersinia pseudotuberculosis IP 32953

Yersinia pseudotuberculosis YPIII

Zymomonas mobilis subsp. mobilis ZM4

uncultured methanogenic archaeon RC-I

Total number of species: 714

The data were downloaded from the NCBI database on June 17, 2008.
