# Supplementary material for: Bacterial Genes in the Aphid Genome: Absence of Functional Gene Transfer from Buchnera to Its Host
Source: PLoS Genet. 2010 Feb 26;6(2):e1000827. doi: 10.1371/journal.pgen.1000827 (PMC2829048; doi:10.1371/journal.pgen.1000827)
Supplement: Text S1 — Screening based on BLASTX. (0.02 MB DOC) [file pgen.1000827.s009.doc]

**Text S1.**

**Screening based on BLASTX**

First, the genome assembly of *A. pisum* (Acyr_1.0) was divided into lengths of 1,000 nucleotides (nts), overlapping by 200 nts. This yielded 585,720 sequences totaling 576,890,191 nts. Sequences with > 50% Ns were removed, leaving 564,560 sequences in the data set (Figure S2, box 1). These sequences were screened by BLASTX (-e 1 -F "m S") against a custom database containing proteomes of 714 bacterial and archaeal species (Table S2), along with the proteomes from the insects *Drosophila melanogaster*, *Anopheles gambiae,* *Apis mellifera*, and *Tribolium* *castaneum* (Figure S2, box 2). This search produced 6,855 sequences in which the top two hits were to bacterial proteins. These sequences were subjected to an additional BLASTX search (-e 1e-2 -F "m S") against the NCBI non-redundant (nr) protein database (Figure S2, box 3). The 530 sequences in which the top three BLASTX hits were to bacterial proteins and that were derived from scaffolds greater than 5,000 nts in length were considered to be preliminary candidates. Among them, 198 sequences were excluded from further analyses (Figure S2, box 4), because these sequences were derived from scaffolds inferred to be bacterial contaminants (see main text, Figure 1, box 5). Manual inspection of the BLAST hit sequences for the remaining 332 sequences revealed that, in 269 sequences, aligned regions were short and only weakly similar to bacterial sequences, suggesting that the results were not reliable. To be conservative, these sequences were also discarded (Figure S2, box 5).

61 of the 63 remaining LGT-candidate sequences appeared to be parts of *A. pisum* proteins predicted by the NCBI and IAGC. Using full-length amino acid sequences of 61 corresponding proteins as query sequences, BLASTP searches were performed against nr protein database at NCBI (Figure S2, box 6). 49 of the 61 proteins were more similar to animal proteins than to bacterial proteins. They were orthologs of proteins widely distributed both in prokaryotes and eukaryotes. Only a part corresponding to each sequence showed slightly higher (BLAST score difference < 13) similarity to bacterial proteins than to animal proteins. Thus, none of these 49 proteins appeared closer to bacterial proteins than to animal proteins, so they were also removed from the LGT-candidates. Finally, 14 sequences corresponding to 11 genes were judged as promising LGT candidates. Ten out of 11 candidates were also detected in our PPP-based screening (Figure S2.7). We thus found one additional candidate (ψ*AtpH*) based on this method.
