# Supplementary material for: Bacterial Genes in the Aphid Genome: Absence of Functional Gene Transfer from Buchnera to Its Host
Source: PLoS Genet. 2010 Feb 26;6(2):e1000827. doi: 10.1371/journal.pgen.1000827 (PMC2829048; doi:10.1371/journal.pgen.1000827)
Supplement: Text S2 — Relative rate test for the A. pisum ψDnaE. (0.03 MB DOC) [file pgen.1000827.s010.doc]

**Text S2.**

**Relative rate test for the *A. pisum* ψ*DnaE***

A long branch leading to the *A. pisum* ψ*DnaE* in the phylogenetic tree suggested that the evolutionary rate of this gene was highly accelerated after the divergence from *Buchnera* *dnaE* (Figure 3). To confirm this observation, we conducted a relative rate test of *K*_A_ and *K*_S_ values between the *A. pisum* ψ*DnaE* and *Buchnera* *dnaE*s, and also performed a functionality test in the lineage of the aphid ψ*DnaE* based on the *K*_A_/*K*_S_ values. The evolutionary rate at non-synonymous substitution sites (*K*_A_) of the *DnaE* in the *A. pisum* lineage (K1 = 0.103) was 3.5 times higher than that in the lineage of *Buchnera* str. APS (K2 = 0.030) (P = 0.008). This suggests that the laterally transferred *dnaE* has rapidly accumulated non-synonymous substitutions. In contrast, the evolutionary rate at synonymous substitution sites (*K*_S_) of the *DnaE* in the *A. pisum* lineage (K1 = 0.041) was 11.7 times lower than that in the lineages of *Buchnera* str. APS (K2 = 0.478) (P= 0.037). This suggests that the mutation rate of the laterally transferred *dnaE* was significantly decreased, possibly to the lower mutation rate in the aphid nuclear genome, after LGT. The *K*_A_/*K*_S_ value of the *A. pisum* ψ*DnaE* lineage (2.5) was not significantly larger than 1, whereas that of the *Buchnera* *dnaE* lineage (0.06) was significantly lower than 1 (P = 0.009). These results, combined with the truncation of the gene, indicate that the aphid ψ*DnaE* is a pseudogene.

**Method for the relative rate test**

Synonymous substitutions per site (*K*_S_) and non-synonymous substitutions per site (*K*_A_) were calculated as described previously (Miyata and Yasunaga, 1980). Multiple substitutions were corrected by Kimura’s two parameter method (Kimura, 1980). Statistical significance of calculated *K*_A_/*K*_S_ values was tested against a bootstrap distribution of *K*_A_/*K*_S_ values, which was generated by 10,000 bootstrap resamplings of codons from the original alignment. Statistical significance of relative rate tests was also inferred by the bootstrap method (Adachi and Hasegawa, 1992). In the relative rate tests, the *dnaE* gene sequences of *Buchnera aphidicola* str. APS (NC_002528) and *Buchnera* str. *Schizaphis graminum* (NC_004061) were used as a functional ortholog and an outgroup, respectively.

**Reference for the relative rate test**

Adachi, J. and Hasegawa, M. (1992) Amino acid substitution of proteins coded for in mitochondrial DNA during mammalian evolution. *Jpn J Genet,* **67:** 187-97.

Kimura, M. (1980) A simple method for estimating evolutionary rates of base substitutions through comparative studies of nucleotide sequences. *J Mol Evol,* **16:** 111-20.

Miyata, T. and Yasunaga, T. (1980) Molecular evolution of mRNA: a method for estimating evolutionary rates of synonymous and amino acid substitutions from homologous nucleotide sequences and its application. *J Mol Evol,* **16:** 23-36.
